# Using digital technology to support wellbeing and independence among people living with incurable cancers: a systematic review

**DOI:** 10.1007/s00520-025-09759-1

**Published:** 2025-07-18

**Authors:** Jordan Curry, Cristina M. Caperchione, Sarah Greenley, Elizabeth Dennis, Cynthia C. Forbes

**Affiliations:** 1 Wolfson Palliative Care Research Centre, Hull York Medical School, University of Hull, Kingston-Upon-Hull, UK; 2 The Activity and Nutrition in Cancer Research Group, Hull York Medical School, University of Hull, Kingston-Upon-Hull, UK; 3 School of Sport, Exercise and Rehabilitation, Human Performance Research Centre, University of Technology Sydney, Sydney, Australia; 4 Hull York Medical School, University of Hull, Kingston-Upon-Hull, UK

**Keywords:** Digital interventions, Incurable cancer, Wellbeing, Feasibility, Physical activity, Exercise, Acceptability

## Abstract

**Purpose:**

The purpose of this systematic review is to summarise and evaluate the feasibility, acceptability, and potential efficacy of using digital technology to deliver physical activity and/or nutrition interventions to promote wellbeing and independence among adults with advanced or incurable cancer.

**Methods:**

Systematic structured searches for any experimental study exploring physical activity and/or nutrition intervention delivery with digital technology were conducted in PsycINFO, MEDLINE, EMBASE, CINAHL, Web of Science Core Collection, and the Cochrane Central Register of Controlled Trials. All records were screened, extracted, and quality assessed by two authors. Main outcomes were feasibility and acceptability of using technology to help deliver interventions, with secondary outcomes of potential efficacy in any measure of quality of life, wellbeing, or function.

**Result:**

Twenty-nine eligible studies were included. Digital interventions were mostly feasible and acceptable, with high retention rates and participant satisfaction. Many participants expressed willingness to recommend the interventions to others or continue use. Engagement rates were generally high, although fewer studies addressed diet and nutrition than exercise and physical activity interventions.

**Conclusion:**

Digital supportive care interventions may be feasible, well-accepted, and tolerated by individuals with incurable cancer. These platforms could effectively improve this population’s support for physical activity and symptom management. However, the heterogeneity in study designs highlights the exploratory nature of these interventions. To advance the field, future research should focus on adequately powered studies, improved generalisability, and standardised tools for measuring outcomes.

**Trial registration:**

This trial has been prospectively registered in PROSPERO (ID: CRD42021295936).

**Supplementary Information:**

The online version contains supplementary material available at 10.1007/s00520-025-09759-1.

## Background

People with incurable or late-stage cancer often deal with significant physical concerns and side effects. This can include increased fatigue, muscle atrophy, pain, excessive weight loss, and chronic nausea and vomiting [1]. This can greatly impact physical conditioning and function, impacting one’s ability to care for themselves, in turn, reducing psychosocial health and quality of life (QoL) [2, 3]. It is important to maintain good physical function to cope with cancer treatments and to be able to live independently. Healthy lifestyle behaviours like being physically active and eating well are known to mitigate cancer-related side effects, improve physical function, and therefore, help improve overall wellbeing among people living with cancer [4, 5].

A recent systematic review assessed the benefits of physical activity (PA) for patients with advanced cancer and reported improvements in physical performance and reduced fatigue [4]. They concluded that PA for those with advanced cancer was safe and feasible and should be integrated as part of usual supportive care. Other research has also reported that PA as an adjuvant therapy has been associated with lower stress, anxiety and depression, as well as improvements in pain, constipation, and insomnia [6], all of which have significant impacts on QoL. Additionally, malnutrition, common among those with late-stage cancers, highlights the critical need for nutrition interventions that address cachexia, a condition of disease-related weight loss and muscle wasting [7]. For best effect, it is recommended that nutrition interventions are combined with PA to maintain physical function and QoL [8, 9].

Despite these benefits, PA and nutrition interventions are rarely incorporated into standard cancer care pathways. If cancer care practitioners recommend these among people with advanced/incurable cancer, engagement is often low [10, 11]. Although this is a trend throughout all cancer stages, low engagement and participation in healthy lifestyle behaviours are more prevalent among those with incurable cancer. This is partly due to specific barriers faced by this cancer population (i.e. safety, loss of appetite/malnourishment, and increased fatigue) as well as access/opportunity to appropriate programmes and services [10, 12–14]. Innovative interventions that address these barriers and are tailored to the needs and preferences of those with advanced/incurable cancers could have a significant impact on improving their physical function and QoL. Recently, using digital technologies to support cancer care has increased in popularity due to its accessibility, scalability, and cost-effectiveness [15–18]. Digital health technologies may include smartphone applications, wearable devices (e.g. Fitbit and Apple watches), web-based tools and programmes, smart home devices (e.g. virtual assistants and motion sensors), and telehealth and video-conferencing software. Studies have shown that telehealth and self-monitoring with wearable activity trackers increase PA, nutrition, and QoL among cancer survivors [17, 19]. Leveraging these tools could improve reach, access, and opportunity for PA and nutrition-based programmes [19]. Beyond providing greater access to services, digital health technologies also provide easily accessible health education and opportunities to connect with others in similar circumstances, providing an additional level of social support.

The benefits of digital health technologies to support access and opportunity to PA and diet interventions for those with cancer are promising. However, those with incurable cancer continue to be an underserved population group with comparatively little research. This review’s purpose is to summarise current evidence and evaluate the feasibility, acceptability, and potential efficacy of digital interventions supporting PA and nutrition behaviours to promote wellbeing and independence among adults living with advanced or incurable cancer. In this review, “wellbeing” refers to multidimensional aspects of QoL, including physical, emotional, and psychosocial domains while independence encompasses the ability to maintain physical function, perform daily activities, and be active autonomously; both essential priorities in advanced cancer care, where the focus often shifts to optimising QoL and personal agency.

## Methods

This review is reported according to the Preferred Reporting Items for Systematic Reviews and Meta-Analyses (PRISMA) guidelines [20] and has been prospectively registered in PROSPERO (ID: CRD42021295936). See Supplement One for PRISMA checklist.

### Search strategy and eligibility criteria

Six electronic databases were searched, including PsycINFO, MEDLINE All, EMBASE (all via OVID), CINAHL via EBSCOhost, Web of Science Core Collection, and the Cochrane Central Register of Controlled Trials (CENTRAL). Development of a search strategy, adapted for each database, was completed with an Information Specialist (SG) and combined three concepts: incurable cancer AND digital technologies AND exercise/diet/lifestyle. The initial search was conducted in December 2021, with the final update search on 9 May 2025. We also performed forward and backward citation searching on the included studies identified from the original search via Citation Chaser on 1 December 2023, removing duplicates and non-cancer-related citations before screening. See Supplement Two for full search development and strategies.

### Study selection

All articles identified through the database searches and citation searches were exported to EndNote 20, duplicates removed, then remaining records uploaded to Covidence systematic review software (Veritas Health Innovation, Melbourne, Australia. Available at www.covidence.org) which was used for title/abstract and full-text screening. Screening was conducted independently by four authors (CF, CMC, JC, SG), requiring two to screen every article against the eligibility criteria (Table 1). Any conflicts were discussed by the respective screeners and resolved by a third author if needed.

**Table 1:** Eligibility criteria

Include	Exclude
(1) Adults (18 years and older) with;	(1) The sample was not comprised solely of incurable cancers or if mixed sample, data presented without individualised cancer data
(2) An incurable cancer looking at the;	(2) The articles were not in English
(3) Feasibility, acceptability, and/or efficacy of using;	(3) The full text articles were not available or
(4) Digital technologies (e.g., web-based, smartphones, wearables, smart home or home-based medical sensors, etc.) to;	(4) They targeted children/adolescents
(5) Support wellbeing and/or independence (e.g. measured by quality of life, life satisfaction, physical function, and performance status) with;	
(6) An intervention focused on nutrition or diet, physical activity or exercise, sedentary behaviour, or mindfulness-based movement practices (e.g. yoga, tai chi, and qigong);	
(7) Published in English	

### Data extraction and quality assessment

Data was extracted using a form based on the Cochrane EPOC template [21], previously piloted by the authors [22]. The authors (ED and JC) independently piloted the form on four studies. Following discussions of discrepancies, the form was finalised. All included studies were independently extracted by two authors (ED, JC, or CF), with any disagreement resolved through discussion. One disagreement occurred, and consensus was resolved via discussion.

Extracted data included study design, setting, main objective, cancer type, intervention, and participant characteristics. Outcomes aligned with the review aims: feasibility (e.g., recruitment, retention, and costs), acceptability (e.g., adverse events, adherence, satisfaction, and engagement), and efficacy (e.g., QoL, breathlessness, physical function, nutrition, and mood).

Methodological quality was assessed independently by two authors (JC, CF, or CMC) using the Standard Quality Assessment Criteria for Evaluating Primary Research Papers from a Variety of Fields [23], which accommodates various study designs. Scores > 80% were rated strong, 71–79% good, 50–70% adequate, and < 50% poor. Discrepancies were resolved through discussion or, when necessary, a third reviewer (JC or CF). All primary articles were appraised; supplemental articles regarding the primary article were used to support findings or provide context and were not subject to quality appraisal.

## Results

### Study selection

**Fig. 1:**
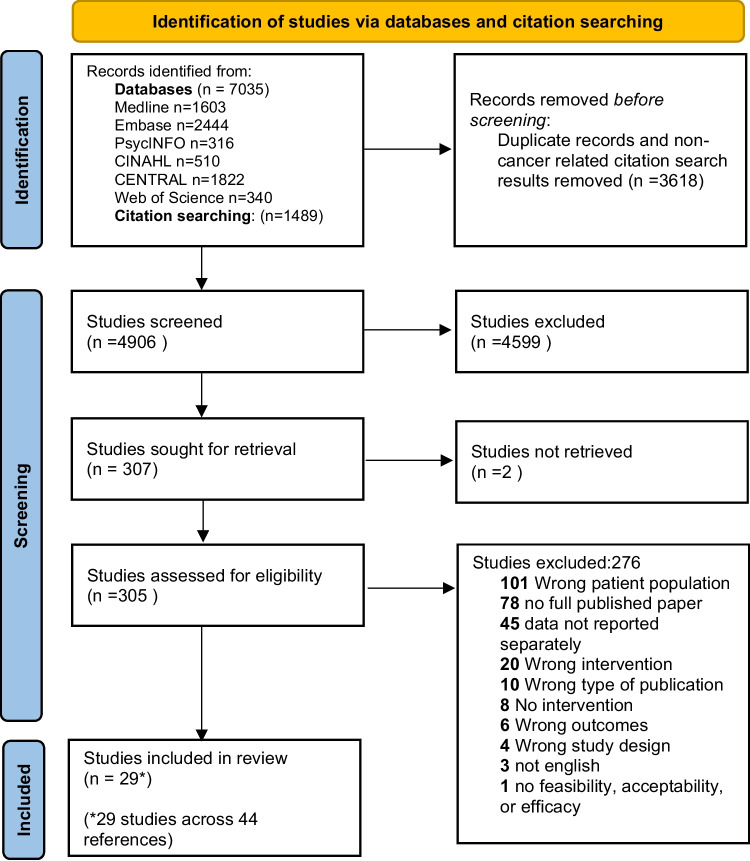
PRISMA diagram: the article identification process

The article identification process is detailed in Fig. 1. Database and citation searches identified a total of 8524 records, of which 4906 remained after de-duplication and removal of non-cancer citations. Following title and abstract screening, 307 studies remained for full-text screening. Multiple reports of relevant studies meant 29 studies (over 44 references) were deemed eligible and were fully extracted.

### Quality appraisal

Twenty-nine studies were appraised for quantitative methods. Twenty-four studies were rated as strong [24–47], three as good [40, 48, 49], one as adequate [50], and one as poor [51]. See details in Supplemental Three4.

### Study characteristics

The types of interventions ranged from PA-focused (*n* = 19) to monitoring QoL and symptoms (*n* = 6), education (*n* = 1), PA and nutrition (*n* = 2), and nutrition (*n* = 1). See Table 2 for full details.

**Table 2:** Study characteristics

Source	Sample size; age	Location	Cancer type	Type of study; duration	Study aim	Intervention description
Asensio-Cuesta et al. (2024)	*n* = 2 Age: P1 (age range 50–55) and P2 (age range 70–75)	Spain	Non-small cell lung cancer	Feasibility/pre-pilot. Single arm	To present and test the Lalaby App to monitor lung cancer patients’ QoL	A 6-week self-guided intervention using the Lalaby app and mobile phone sensors
Bade et al. (2018)	*n* = 35; mean 66	USA	Lung cancer	Comparative study of two non-randomised trials	To test that a PCAR will improve patient participation and physical activity more effectively than weekly phone calls	A 4-week programme using Fitbit devices, combining self-guided walking with researcher phone calls or education/text-based motivation
Bade et al. (2021)	*n* = 40; mean 65	USA	Non-small cell lung cancer	Open-label, pilot two arm RCT	A Pilot study to determine interest in participating in a 3-month home-based walking programme	A 12-week intervention using Fitbit Flex2, with in-person clinician teaching and framed text messages, followed by independent activity
Bergerot et al. (2025)	*n* = 41; mean 73.4; median 70	Brazil	Mixed Advanced Cancers	Pilot, single arm	To assess the impact of a 12-week supervised remote exercise programme on the health-related QoL of older patients with cancer undergoing systemic cancer treatment in Brazil	A 12-week supervised, remote programme using WhatsApp and online video sessions
Cheong et al. (2018)	*n* = 75; mean 58	South Korea	Colorectal	Single arm, interventional trial	To evaluate the efficacy and feasibility of comprehensive mobile health care using a tailored rehabilitation programme	A 12-week self-guided intervention using an app and IoT wearable for activity and symptom tracking
Cheville et al. (2012)	*n* = 66; mean 64.7	USA	Lung and colorectal	RCT, 2 arm	To conduct a trial of a home-based exercise intervention that can be integrated into established delivery and reimbursement structures	An 8-week programme with pedometer tracking, initial physio session, and bi-monthly clinician phone calls
Cheville et al. (2019)	*n* = 516; mean 65.6	USA	Mixed advanced cancers	RCT, 3 arm	To determine whether collaborative telerehabilitation and pharmacological pain management improve function, lessen pain, and reduce requirements for inpatient care	A 6-month web-based programme with clinician-led exercise components
Coats et al. (2020)	*n* = 5; mean 62	Canada	Thoracic neoplasia	Single-arm pilot study	To evaluate if a home-based exercise programme (TELERP) with real-time monitoring can improve physical functioning in patients with unresectable thoracic neoplasia undergoing chemotherapy	An 8-week hybrid telerehabilitation using videoconferencing, sensors, and webcam, including supervised and self-led sessions
Crosby et al. (2023)	*n* = 11; mean 61.6	Australia	Stage III–IV melanoma	Non-randomised feasibility pilot trial	To determine the feasibility, safety and preliminary efficacy of a telehealth supervised exercise programme	An 8-week virtual, clinician-led programme delivered via Zoom
Delrieu et al. (2020)	*n* = 49; mean 55	France	Metastatic breast cancer	Single arm feasibility study	To assess the feasibility of a physical activity intervention, on functional, psychological, and clinical parameters	6-month self-directed programme using a Nokia Go wristband and periodic instructor phone calls
Dorion et al. (2017)	*n* = 12; mean 62	Canada	5 prostate, 3 breast, 3 lung, 1 colon	Prospective cohort pilot study embedded within a larger RCT	To determine the feasibility of using AT to assess the patient prognosis and the effects of palliative RT	Short-term pre/post radiotherapy activity monitoring using the Misfit Flash tracker
Evans et al. (2021)	*n* = 40; mean 70.2	Australia	Metastatic prostate cancer	Pilot RCT	To explore the acceptability, safety and preliminary efficacy of a web-based exercise intervention (ExerciseGuide)	An 8-week self-guided intervention delivered via a computer-tailored website
Hacker et al. (2020)	*n* = 34; mean 62.3	USA	Multiple myeloma	Pilot RCT	To assess the feasibility, acceptability, and effects of a physical activity intervention (STEPS) vs. UC on activity levels, fatigue, strength, function, sleep, and QoL after HCT for MM	A 6-week mixed approach using physical activity trackers and clinician-led education followed by independent activity
Kenfield et al. (2021)	*n* = 25; median age 71 years	USA	Metastatic castrate-resistant prostate cancer	Pilot RCT, 3 arm-trial	To report on feasibility, safety, acceptability, cardiovascular fitness, strength, and Halabi prognostic score outcomes	A 12-week clinician-led remote monitoring programme using Polar HR strap and app
Keum et al. (2021)	*n* = 40; median age 61.5	South Korea	Pancreatic ductal adenocarcinoma	RCT	To evaluate the efficacy of a mobile app–based programme, Noom, in patients receiving chemotherapy for PDAC	A 12-week self-guided app programme with clinician feedback via the app
Kim et al. (2018)	*n* = 76; mean 50.9	Republic of Korea	Breast	RCT	To evaluate if a mobile game-based education can improve drug compliance, reduce chemotherapy side effects, and enhance psychological wellbeing in breast cancer patients	A 3-week self-directed mobile game-based intervention
Lee et al. (2024)	*n* = 37; mean 63.3	USA	CLL	RCT (randomised factorial design)	To develop a feasible and effective multi-component lifestyle intervention for patients with CLL, we conducted a pilot study, HEALTH4CLL, using a multiphase optimization strategy (MOST)	A 16-week self-directed intervention using a mobile phone and Fitbit
Longacre et al. (2020)	*n* = 516; mean 65.6	USA	Mixed advanced cancers	Secondary analysis of COPE trial (RCT)	To evaluate the cost-effectiveness of a centralised telecare approach using CCM for delivering rehabilitation to late-stage cancer patients with functional limitations	A 6-month tele-rehabilitation programme using a pedometer and clinician-led telehealth sessions
Low et al. (2023)	*n* = 26; mean 56.2	USA	Metastatic GI/peritoneal malignancy	Pilot RCT	Evaluate the feasibility and preliminary effects of a perioperative SB intervention on activity behaviour, quality of life, symptoms and 30-day readmissions	A self-directed programme using smartwatches and phones from pre-surgery through 30-days post-discharge
Park et al. (2019)	*n* = 100; mean 55.1	South Korea	Advanced lung cancer	Prospective single-arm intervention pilot study	To determine the feasibility and efficacy of smartphone app–based PR on exercise capacity, symptom management, and QoL	A 12-week primarily self-directed programme using a smart aftercare app and IoT wearable
Phillps et al. (2024)	*n* = 49; mean 54.8	USA	Metastatic breast cancer	RCT	Examine the feasibility of a tailored, theory-guided, 12-week mHealth intervention to promote activity of any intensity via increasing daily steps in MBC patients	A 12-week hybrid model using Fitbit and app with weekly clinician calls
Purdy et al. (2022)	*n* = 28; mean 65	Canada	Multiple myeloma	Single-arm feasibility study	To assess the safety, feasibility, and preliminary efficacy of a 12-week virtual eHealth exercise programme in people with MM	A 12-week programme offering supervised virtual group workouts alongside independent exercises
Schmitz et al. (2021)	*n* = 17; mean 60	USA	Metastatic breast cancer	Pilot RCT	A pilot study that aimed to further evaluate acceptability, feasibility, patient satisfaction, cost, and initial efficacy of the Nurse AMIE tablet-based supportive care platform	A 3-month app-based programme combining self-directed use with weekly navigator phone support
Schmitz et al. (2023)	*n* = 42; mean 53.3	USA	Metastatic breast cancer	RCT with partial crossover	To test a virtual assistant for addressing symptoms in MBC using the Amazon Echo Show with Alexa	A 6-month self-directed intervention using Amazon Echo Show with Alexa
Shachar et al. (2023)	*n* = 52; median 55	USA	Metastatic breast cancer	Single-arm pilot study	To explore recruitment, retention, and potential benefits to QoL and function from a home-based walking intervention in women undergoing treatment for MBC	A 3-month self-directed programme using Fitbit Zip
Soh et al. (2018)	*n* = 203; gastric: < 40: 12 (11.9%), 40 s: 24 (23.8%), 50 s: 37 (36.6%), 60 s: 22 (21.8%), > 70: 6 (5.9%). Colon: < 40: 4 (3.9%), 40 s: 15 (14.7%), 50 s: 35 (34.3%), 60 s: 35 (34.3%), > 70: 13 (12.7%)	Korea	Colon and gastric cancer	Interventional observation study	To develop and validate a multidisciplinary mobile care system with self-monitoring features that can be useful for patients with advanced gastrointestinal cancer	A 12-week self-guided mobile health app-based intervention
Wallace et al. (2025)	Advanced *n* = 84; Mean CL: 50.89; Mean CM 52.35	Australia, USA, New Zealand, UK, and Canada	Mixed	RCT	Assess and compare the efficacy of an emotion-focused (CanCopeMind [CM]) and lifestyle (CanCopeLifestyle [CL]) intervention to improve HRQoL among cancer survivors	An 8-week web app programme with self-directed learning modules
Wang et al. (2021)	*n* = 4; mean 63.3	USA	Mixed advanced cancers: breast, head and neck, melanoma, rectal	Pre-efficacy phase testing (Phase I and Phase IIa trials)	To evaluate the feasibility, acceptability, and safety of PAfitME™ and examine intervention targets, risk factors, and clinical outcomes	A 6-week independently conducted programme using Wii Fit
Wolff et al. (2024)	*n* = 26; median: 52.5 (IG), 54.5 (CG)	Germany	MBC	RCT	The present study was designed to demonstrate the medical benefits and positive care effects of the app in terms of psychological distress among breast cancer patients in a multicentric setting to substantiate and validate the results of the pilot study	A 12-week self-directed intervention delivered via a mobile app

*PA* Physical activity, *IG* intervention group, *UC* usual care, *MBC* metastatic breast cancer, *SB* sedentary behaviour, *PR* pulmonary rehabilitation, *MM*  multiple myeloma, *HCT* haematopoietic cell transplantation, *DASH*  detecting activity to support healing, *CCM*  collaborative care model, *PDAC* pancreatic ductal adenocarcinoma, *AT* activity trackers, *RT* radiation therapy, *TELERP* telerehabilitation programme, *PCAR* patient-centred activity regimen, *IoT* Internet of Things, *QoL* quality of life, * RCT* randomised controlled trial, *HR* heart rate, *REST* rapid, easy, strength training exercises, *CG *control group, *6MWT* 6-min walking test, *RPE* Rating of Perceived Exertion Scale, *CLL* chronic lymphocytic leukaemia

Eight studies were randomised controlled trials (RCTs) [26, 33, 34, 44–48], seven pilot RCTs [25, 30–32, 36, 43, 49] and an RCT with one partial crossover [38]. Eight studies used a single-arm pilot or feasibility design [24, 27–29, 37, 39, 51, 52], with three using comparative or observational methods [40, 42, 50], one secondary analysis of an RCT [35], and one prospective cohort study embedded within a larger RCT [41].

The total combined sample size consisted of 2391 participants. Twenty-four studies were conducted in six countries: the USA (*n* = 14) [25, 26, 31, 32, 35, 36, 38–40, 44, 45, 48–50], Korea (*n* = 5) [33, 34, 37, 42, 51], Canada (*n* = 3) [27, 41, 52], Australia (*n* = 2) [28, 30], France (*n* = 1) [29], Brazil (*n* = 1) [43], Spain (*n* = 1) [24], Germany (*n* = 1) [47], and one multinational study, covering Australia, the USA, New Zealand, the UK, and Canada [46].

Eight studies had mixed cancer populations [26, 35, 40–43, 46, 48], followed by seven breast [29, 34, 38, 39, 45, 47, 49], four lung [24, 25, 37, 50], two prostate [30, 32], two multiple myeloma [31, 52], and single cases for colorectal [51], thoracic neoplasia [27], melanoma [28], pancreatic [33], chronic lymphocytic leukaemia [44], and gastrointestinal [36].

The technology used included wearables (*n* = 7) [25, 29, 31, 39, 41, 48, 50], applications (*n* = 6) [33, 34, 42, 47, 49, 52], wearables and applications (*n* = 7) [24, 32, 36, 37, 44, 45, 51], application and video (*n* = 1) [43], or Alexa (*n* = 1) [38], websites (*n* = 3) [26, 30, 46], telecommunication/telerehabilitation systems (*n* = 2) [28, 35], combination of telerehabilitation and wearables (*n* = 1) [27], and a Wii (*n* = 1) [40]. Interventions varied widely in delivery mode, duration (ranging from three to 26 weeks), and level of guidance. While many were primarily self-guided, several incorporated clinician-led components such as virtual supervision, education sessions, or regular phone support. Interventions commonly used mobile apps, fitness trackers, or web platforms to provide real-time feedback, activity monitoring, and behavioural support aimed at enhancing physical activity or recovery outcomes.

#### Feasibility

Nineteen of the 29 studies provided information regarding the recruitment rate [25, 26, 28–36, 38, 40, 43–45, 48, 49, 52]. Mean recruitment rate was 62%, and the median was 66%. Eleven studies reported recruitment targets [25, 26, 28, 30, 32, 33, 35, 39, 40, 49, 52], of which seven met their recruitment target [25, 26, 33, 35, 39, 49] and four did not [28, 30, 32, 40]. The mean retention rate (*trial retention, defined as the proportion of participants who completed follow-up assessments relative to those enrolled at baseline*) of those reporting this (*n* = 27) was 85%, and the median was 87% [24–45, 48–52].

Two studies considered cost-effectiveness [35, 49]. Longacre et al. (2020) concluded that telerehabilitation was more cost-effective than either enhanced usual care or telerehabilitation combined with pain management, with the latter being a dominated strategy (*i.e. more costly and less effective*). Schmitz et al. (2021) reported intervention costs of $570.23, excluding software development and additional healthcare costs. Cheville also reported on additional hospital-related outcomes, including days hospitalised, length of stay, discharge to home, and planned admissions for treatment. In this study, telerehabilitation was associated with higher odds of home discharge, fewer hospital days, and reduced hospital stay [26]. Further details are provided in Table 3.

**Table 3:** Feasibility outcomes

Source	Recruitment rate	Retention rate (%)	Recruitment target met	Financial cost of implementation
Asensio-Cuesta et al. (2024)	NM	100%	NM	NM
Bade et al. (2018)	NM	75.86%	NM	NM
Bade et al. (2021)	56%	97.5%	Yes	NM
Bergerot (2025)	85.42%	72.2%	NM	NM
Cheong et al. (2018)	NM	73.5%	NM	NM
Cheville et al. (2012)	87%	85%	NM	NM
Cheville et al. (2019)	6.6%	85.6%	Yes	Hospitalisations (*n*): 45 (Arm 1), 61 (Arm 2), 57 (Arm 3) Days hospitalised (*n*): 335 (Arm 1), 213 (Arm 2), 284 (Arm 3) Length of stay M(SD), 95% CI: 7.4 (9.3), 4.7–10.2 (Arm 1) vs. 3.5 (4.3), 2.4–4.6, *p* = 0.01, Arm 1 vs. 5.0 (7.2), 3.1–6.9, *p* = 0.18 Discharge to home *n* (%): 20 (44.4%) (Arm 1), 45 (73.8%) (Arm 2), 41 (71.9%) Arm 3 Arm 1 vs. Arm 2: OR (95% CI), *p*: 4.3 (1.3–14.3), *p* = 0.02 Arm 1 vs. Arm 3 OR (95% CI), *p*: 3.8 (1.1–12.4), *p* = 0.03 Planned admission for treatment *n* (%): 8 (17.8%) Arm 1, 24 (39.3%) Arm 2, 17 (29.8%) Arm 3 Arm 1 vs. Arm 2: OR (95% CI), *p*: 6.3 (0.9–45.8) *p* = 0.06 Arm 1 vs. Arm 3: OR (95% CI), *p*: 1.6 (0.3–8.8) *p* = 0.62
Coats et al. (2020)	NM	100%	NM	NM
Crosby et al. (2023)	48%	91%	No	NM
Delrieu et al. (2020)	94%	90%	NM	NM
Dorion et al. (2017)	NM	75%	NM	NM
Evans et al. (2021)	28.37%	92.5%	No	NM
Hacker et al. (2020)	76%	94%	NM	NM
Kenfield et al. (2021)	5.5%	83.3%	No	NM
Keum et al. (2021)	83%	82.5%	Yes	NM
Kim et al. (2018)	91.5%	94.7%	NM	NM
Lee et al. (2024)	61.4%	83.8%	NM	NM
Longacre et al. (2020)	38.3%	97.4%	Yes	Mean incremental cost of telerehabilitation (Arm B) over enhanced usual care (Arm A): $154.94 per patient Mean effectiveness gain: 0.01 Quality-Adjusted Life Years (QALYs) Incremental cost-effectiveness ratio (ICER) for telerehabilitation (Arm B): $15,494 per QALY Arm C was more costly and less effective than Arm B, making it not cost-effective. At a $100,000 willingness-to-pay threshold, Arm B was cost-effective in 95.4% of simulations, while Arm A was cost-effective in 4.6%. Telerehabilitation (Arm B) remained cost-effective at thresholds as low as $15,494
Low et al. (2023)	74%	88%	NM	NM
Park et al. (2019)	NM	90%	NM	NM
Phillips et al. (2024)	21.12%	100%	NM	NM
Purdy et al. (2022)	93.5%	90%	Yes	NM
Schmitz et al. (2021)	68%	One-month:76% Three-month: NR	Yes	$570.23 per patient (not including cost of software development or patient healthcare cost)
Schmitz et al. (2023)	51%	88%	NM	NM
Shachar et al. (2023)	NM	77%	Yes	NM
Soh et al. (2018)	NM	86.7%	NM	NM
Wallace et al. (2025)	NM	NM	NM	NM
Wang et al. (2021)	17%	40%	No	NM
Wolff et al. (2024)	NM	NM	NM	NM

*NR* Not reported, *NM* not measured*,* *Arm 1 *automated monitoring,  *Arm 2* telerehabilitation, *Arm 3* telerehabilitation with pain management

#### Acceptability

Engagement with a device and associated online content was high across all studies. Most participants actively engaged with the online components of the interventions, consistently accessing the provided material and participating in the recommended PA and nutrition. However, a gradual decline in engagement was noted, particularly in studies with a primarily educational focus [30, 36, 38]**.**

Adverse events were reported in 14 studies [25, 27, 30, 32–34, 39, 41, 48, 49, 51, 52]. Commonly reported issues included hospitalisations for pre-existing conditions, occasional mild symptoms like dizziness, and some musculoskeletal pain, which were generally well managed [30, 32, 51, 52]. Cheville et al. (2019) reported significant differences among arms for frequency and duration of hospitalisations [26]. All adverse events were minor, unrelated to interventions, and had minimal impact on adherence.

Overall, participant satisfaction with the digital technology part of interventions was high, with nine studies [24, 25, 34, 37, 38, 43–45, 50] reporting that participants would recommend or continue using the tool. Another ten studies [26, 27, 30, 32, 36–38, 40, 52] highlighted general satisfaction with the intervention.

Common concerns reported included usability difficulties (e.g., difficulty handling) [34, 37] and frequent system errors [37]. One study included suggestions for enhancing participant experience, including increasing PA variety to reduce boredom, improving usability and navigation, and enhancing the frequency of personalised support from exercise physiologists [30]. Details are found in Table 4.

**Table 4:** Acceptability outcomes

Source	Engagement with technology	Participant adverse events	Participation adherence rates	Participant satisfaction
Asensio-Cuesta et al. (2024)	Patient 1: Reported activities/symptoms 47 times in 31 days (irregular, between 10 and 3 reports/week) Recorded 168 activities and 63 symptoms Completed 24 UEQ-S entries Patient 2: Reported activities/symptoms 30 times over 29 days (irregular, 1–4 reports/week) Recorded 83 activities and 89 symptoms Completed 4 UEQ-S entries	NM	Patient 1: Reported EORTC QLQ-C30 51 times Reported ECOG 17 times Completed 24 UEQ-S entries Patient 2: Reported EORTC QLQ-C30 14 times Reported ECOG 8 times Completed 4 UEQ-S entries	Patient 1: Neutral app evaluation (hedonic score, 0.71; pragmatic score, 0.47; global score, 0.59) Positive feedback given, such as “inventive” and “leading edge” Desired app features: symptom reporting over time, quality of life (QoL) tracking, and an agenda for medical consultations Patient 2: Positive app evaluation (hedonic score, 2.5; pragmatic score, 2.68; global score, 2.59) Positive adjectives: “leading-edge”, “inventive”, “interesting”, “exciting”, “clear”, “easy”, and “supportive” Both patients indicated they would recommend the app to other patients. Both would have preferred more feedback from the app
Bade et al. (2018)	Text-message group: 100% (15/15) used the device, with 0% never using it 11% (47/420) days had no step counts 92% found intervention helpful, 75% wanted to continue tracking activity Most preferred receiving text messages once/day (50%) and at noon (58%) Weekly phone call group: 79% (23/29) used the device, 21% never used it 38% (305/812) days had no step counts	NM	Text message group: 100% adherence used the device 11% days had no step count data 75% expressed a desire to continue tracking their activity Weekly phone call group: 79% used the device, 21% never used it 38% days had no step count data	Text message group: 92% found intervention helpful, 85% would participate in another activity study Weekly phone call group: 83% were not interested in participating in group activities 85% would participate in another PA study
Bade et al. (2021)	Intervention group provided usable data for 90% weeks during the study	4 serious adverse events occurred 3 hospitalizations for chronic obstructive pulmonary disease exacerbation, pneumonia, and hyperthyroidism 1 emergency room visit due to a fall 2 minor adverse events reported Ankle pain and bronchitis	Individualised walking goals were met 21% of weeks 100% adhered to the study, no reported dropouts	Intervention group: 85% (17/20) found the intervention helpful 90% (18/20) would participate in a future activity study 95% (19/20) plan to continue walking for exercise 85% (17/20) plan to continue tracking their activity
Bergerot (2025)	26 patients demonstrated great adherence by completing the full 12-week intervention, while 10 patients exhibited moderate adherence, completing eight out of the 12 weeks	No adverse events reported	87.8% adhered to the programme	Most participants (87.8%) reported satisfaction with the programme
Cheong et al. (2018)	NM	No adverse events reported Rate of dizziness and dyspnoea during exercise was < 15%	Compliance of 83% based on weekly survey of compliance	26% of participants withdrew over course of study
Cheville et al. (2012)	NM	More participants died in the intervention group, (5 in the intervention and 2 in the control group, *P* = 0.28)	Mean daily step count increased over the 8-week duration	NM
Cheville et al. (2019)	Frequency of automated monitoring contacts M(SD): Arm 1: 10.3 (4.4) Arm 2 10.7 (5.2) Arm 3 10.2 (4.5) Proportion using web-based instead of interactive voice recognition reporting Arm 1: 66.4% Arm 2: 73.6% Arm 3: 69.1%	None reported	Fitness Care Manager contacts Frequency M(SD), range Arm 2: 7.6 [2.9], 1–21 Arm 3: 7.2 [3.1], 1–22 Duration (minutes) M(SD), range Arm 2: 16.2 (15.2), 1–124 Arm 3: 16.6 (15.4), 1–87 Physical therapy visits M(SD) Arm 2: 5.8 (5.9) Arm 3: 5.2 (8.1)	NM
Coats et al. (2020)	1/75 supervised telerehabilitation sessions had to be delayed due to inability of establishing audiovisual communication between patient and clinician	No major adverse events reported Brief episodes of oxygen desaturation (SpO₂ < 88%) were recorded 10 events during supervised sessions 6 events during unsupervised sessions No severe oxygen desaturation (SpO₂ < 80%) was recorded	All patients 15/15 sessions prescribed supervised exercise sessions Participants completed an average of 96% of prescribed unsupervised exercise sessions Duration (M(SD)) of supervised sessions: 67 (12) min Cardiovascular exercise time per session in minutes, M(SD): Supervised sessions: 18 (6) Unsupervised sessions: 26 (9) Adherence to intensity levels(M% of time): Supervised sessions: 42% low, 37% moderate, and 22% high intensity Unsupervised sessions: 40% low, 37% moderate, and 23% high intensity Resistance training duration M(SD) 13.9 (1.5) min per session	All five patients reported being quite satisfied (score of 4) or very satisfied (score of 5) with the telerehabilitation platform Most important aspects of the platform, according to patients: Ease of use (*n* = 4) Efficacy (*n* = 4) Dimensions of the platform (*n* = 2) Communication system (*n* = 2) Satisfaction scores M(SD) %: Global satisfaction: 87 (12) Satisfaction with: Health care professionals: 82 (14) Services delivered: 90 (14) General health care organisation: 93 (12)
Crosby et al. (2023)	Programme attendance, median (IQR): 87.5% (IQR: 75.0–91.7%). 226/264 prescribed exercise sessions completed	None reported One minor exercise-related AE (surgical wound reopening (calf))	87.6% of sessions were at or above prescribed RPE, 12.4% were completed at a lower RPE than prescribed Completion rate: 91% Median exercise compliance: Resistance exercise, 82.1%; aerobic exercise, 84.9%	NM
Delrieu et al. (2020)	96% of patients met adherence criteria of wearing (at least one week of consecutive wear during the 6-month study)	NM	77% met activity guidelines (≥ 630 MET-minutes/week) at 6-month measures Of the 31 (70%) who met the recommendations at baseline, 29 met the recommendations at 6 months	NM
Dorion et al. (2017)	75% wore the AT before radiotherapy 83.3% of patients wore the AT after treatment	2 deaths unrelated to intervention 1 withdrawal due to pneumonia	Days of recorded steps (median) pre-RT: 3, post-RT: 6 Compared to pre-RT, the patients after palliative RT took 30% less daily steps (*p* < 0.02)	11/12 of participants wearing ATs (good acceptance)
Evans et al. (2021)	Time on website in minutes M(SD), range 93.3 (101.6), 4.3–373.6 Website logins M(SD), range 6.1 (5.9), 1–22 100% completion rate in week 1–3 modules Remaining modules competition rates ranged from 45 to 75% Decrease in completing PA tracking modules over time: 65% week 1, 10% at week 3, 0% at week 8) Telehealth consult time in minutes M(SD): Week 1: 25.15 (7.80) Week 4: 23.92 (7.47) Text Messages Sent: 65 Emails Sent: 81 Phone Calls: 11; average duration 11.50 (8.46) min	Reported bone pain: No bone pain: 85% Mild bone pain: 10% Moderate bone pain: 5% Severity of pain post-exercise M(SD): Bone pain: 0.3 (0.8) post-resistance exercise, 0.4 (0.8) post-aerobic Non-bone pain: 0.7 (0.9) post-resistance, 0.6 (0.9) post-aerobic	Exercise diary completion rate 89% Resistance training session adherence: 64.6% (40.2) Aerobic training session adherence: 102% (62.7) Perceived exercise intensity: 6.6/10 resistance exercise sessions 6.6/10 aerobic sessions Participants rated their self-perceived adherence to their aerobic exercise programme as 6.1/10 and 5.4/10 for their resistance training	Intervention satisfaction: CSQ-8 median (range) 28.0 (16–31), max 32 SUS scale M (SD) 67.0 (15.1) max 100 PESS median scores Overall: 6.5/7.0 Subscores: autonomy, 6.2; structure, 6.6; involvement, 6.2 Website relevance: 6.0/7.0
Hacker et al. (2020)	76% of the STEPS arm wore the physical activity tracker device on more than 90% of the study days 24% wore the physical activity tracker less than 13 days, 2 did not wear it at all	NM	Achieved PA step goals an average of 18.65 (SD, 9.41) out of 35 days	No participants reported wearing the physical activity tracker interfered with their lives 88.2% willing to participate in another PA study wearing a PA tracker All participants would recommend this type of research to a friend. 82.4% planned to continue wearing the PA tracker after study completion
Kenfield et al. (2021)	Remote intervention participants attempted 93% of prescribed workouts Pre–post-exercise session survey completion rate: 92% resistance arm, 94% aerobic arm	Total AEs: 14 reported, 8 in the resistance arm Possibly related AEs: aerobic arm: one hip and lower back pain. Resistance arm: two pain in the heel and shoulder Treatment of pain: two participants received pain medication	Of sessions attempted, 87% reported completed as prescribed or with more sets, reps, and/or weight (88% resistance and 86% aerobic) Survey completion rates: resistance arm, 92%; aerobic arm, 94%	Overall programme satisfaction 90% being satisfied or very satisfied Quality of programme: 90% very good or excellent, 90% reported they would recommend the study to others
Keum et al. (2021)	Meal input frequency (meals per week) M (SD): Noom users (*N* = 17): 11.15 (7.69) Above average users (*n* = 10): 15.41 (6.76) Below average users (*n* = 7): 5.06 (3.95) Exercise input frequency (every 12 weeks) M(SD): Noom users (*N* = 17): 3.35 (8.19) Above average users (*n* = 10): 5.61 (10.28) Below average users (*n* = 7): 0.14 (0.38) Articles read (per week) M(SD): Noom users (*N* = 17): 0.98 (1.84) Above average users (*n* = 10): 1.47 (2.33) Below average users (*n* = 7): 0.27 (0.33) Weight inputs (inputs/week) M (SD): Noom users (*N* = 17): 0.6 (0.72) Above average users (*n* = 10): 0.83 (0.7) Below average users (*n* = 7): 0.27 (0.65) Messages to coach (per week) M(SD): Noom users (*N* = 17): 5.14 (5.56) Above average users (*n* = 10): 6.19 (4.35) Below average users (*n* = 7): 3.63 (7.03)	Severe medical events: 2 patients (1 per group) could not continue due to severe sepsis or disease progression	Meeting minimum protein requirement: Above average users: 70% Below average users: 0% Meeting minimum energy intake requirement: Average users 60% Below average users 0% Attrition rate: 18% (7/40 participants) Reasons for dropout: withdrew consent: 5 patients (2 Noom users, 3 non-Noom users) Follow-up completion rate: Noom users: 17/20 Non-Noom users: 16/20	NM
Kim et al. 2018	Game group: 41% of game played (quests, level ups, and rewards) Time spent in interventions M(SD) in minutes: Game group: 22.2 (6.1) vs self-education: 5.5 (4.0); *p* < 0.001)	Physical adverse events (*n*, %) Nausea: baseline: game: 29 (85%), control: 23 (61%), *p*-value: 0.02. Grade ≥ 3 physical adverse events: baseline: game: 5 (15%), control: 0 (0%),* p*-value: 0.02 Fatigue: baseline: game: 16 (47%), control: 29 (76%), *p*-value: 0.02. Grade ≥ 3 physical adverse events: baseline: game: 1 (3%), control: 12 (32%), *p*-value: 0.002 Decreased appetite: Baseline: game: 16 (47%), control: 11 (29%), *p*-value: 0.18. grade ≥ 3 Physical adverse events: baseline: game: 3 (9%), control: 6 (16%), *p*-value: 0.59 Numbness of hand/foot: baseline: game: 0 (0%), control: 22 (58%), *p*-value: 0.02. Grade ≥ 3 physical adverse events: baseline: game: 0 (0%), control: 3 (8%), *p*-value: 0.28 Stomatitis: baseline: game: 0 (0%), control: 4 (11%), *p*-value: 0.15. Grade ≥ 3 physical adverse events: baseline: game: 0 (0%), control: 3 (8%), *p*-value: 0.28 Gastrointestinal (diarrhoea or constipation): baseline: game: 7 (21%), control: 9 (24%), *p*-value: 0.97. Grade ≥ 3 physical adverse events: baseline: game: 1 (3%), control: 5 (13%), *p*-value: 0.25 Hair loss: baseline: game: 0 (0%), control: 10 (26%), *p*-value: 0.27. Grade ≥ 3 physical adverse events: baseline: game: 0 (0%), control: 8 (21%), *p*-value: 0.01 Skin rash: baseline: game: 0 (0%), control: 0 (0%), *p*-value: N/A. Grade ≥ 3 physical adverse events: baseline: game: 0 (0%), control: 0 (0%), *p*-value: N/A		56% found it difficult to use 72% were willing to play again 67% of patients found the game fun 61% of patients found the game helpful for taking medications 74.4% appreciated the information provided about breast cancer and treatment 73.9% found the game helpful in overcoming chemotherapy side effects 81% reported they would recommend the game to other patients with breast cancer
Lee et al. (2024)	NM	NM	Completion rates: telephone coaching: 85.7%, email coaching: 81.3%, text message reminders: 84.2%. No reminders: 83.3%, aerobic exercise alone: 90.0%, resistance and aerobic exercise: 76.5%, weekly self-monitoring: 94.4%, daily self-monitoring: 73.7%	Group mean satisfaction scores: telephone coaching: 4.1, email coaching: 3.5, text message reminders: 3.2, self-monitoring (4–7 days/week): 4.4, self-monitoring (1 day/week): 4.2, Fitbit use: 4.2, overall programme satisfaction: 4.3 Participants reported higher satisfaction with: telephone vs. email coaching, text reminders vs. no reminders, resistance + aerobic exercise vs. aerobic alone
Low et al. (2023)	Percent prompts leading to taking steps: 22% (418/1925) post-surgery out of hospital Pre-surgery (49%): mean = 3.3 prompts per day, SD = 1.8 Post-surgery, in hospital (6%): mean = 7.8 prompts per day, SD = 2.6 Post-discharge recovery (18%): mean = 6.2 prompts per day, SD = 2.6 Days completing symptom ratings: 62% Days Fitbits worn: 77% (91% of days had ≥ 8 h of data) Percentage of days ≥ 8 h of Fitbit data ranged from 17% (9/52 days) to 100% (58/58 days) Participants became less adherent with symptom reporting and wearing the Fitbit after surgery, there were no significant differences in adherence between groups	NM	Retention rate: 88% 3 participants withdrew: Reasons for withdrawal included feeling overwhelmed, technology aversion, use of a different device, and poor health post-surgery	Phone and watch interfaces were pleasant and easy to use Overall system satisfaction and usability mean Intervention group: 93.1 Monitoring-only group: 91.6 Enjoyment in tracking activity data (e.g., steps) via Fitbit app; participants set personal recovery goals and observed benefits Overall intervention satisfaction had a mean score of 88.5 and usability 85.1
Park et al. (2019)	The mean exercise number per week was 3.8 (SD 1.2) at 1 week, 4.2 (SD 1.1) at 6 weeks, and 4.1 (SD 1.2) at 12 weeks, satisfying the exercise prescription	No adverse events reported	85/90 patients completed the 6MWT 86/90 completed all questions of EORTC QLQ-C30	Satisfied with service: 77% would recommend to others: 88% All patients reported that the management algorithms for adverse events were helpful for controlling symptoms and determining when to visit the hospital Patients who reported dissatisfaction with the service mostly cited difficulty in handling the app and frequent system error
Phillips et al. (2024)	Fitbit worn on 92.7% of study days (SD = 9.9) Fit2ThriveMB app opened on 94.1% of study days (SD = 17.5) 88.2% said notifications increased app use; 45.5% reported learning something new 72.7% said notifications encouraged activity; 68.2% were motivated to reach step goals	No adverse events reported	Weekly coaching call attendance: 98.3% (SD = 5.4); avg. call length 22.0 min (SD = 15.0) 12-week assessment completion: 98% valid accelerometer wear (*n* = 48), 95.9% questionnaire completion (*n* = 47), 96% functional performance test completion (*n* = 24) Interview completion: 88% (22/25); survey completion: 92% (23/25)	100% were satisfied/very satisfied with the overall experience and study staff Mean likelihood to recommend programme: 8.7/10 90.9% felt coaching calls helped them reach goals Coaching calls rated positively for: frequency (95.5%), timing (100%), duration (76%), coach professionalism (100%), 81.8% were satisfied with technical support 81.8% found Fitbit setup with Fit2ThriveMB easy 86.4% liked Fitbit integration into the Fit2ThriveMB app 86.4% said Fitbit integration increased motivation and goal attainment 86.4% found the Fit2ThriveMB app easy/very easy to use. 68.2% were satisfied with app design; 72.7% with app content 86.4% felt confident using the Fit2ThriveMB app
Purdy et al. (2022)	Completed workouts: 82.9% independent home workouts 89.9% group workouts 89.7% aerobic exercise Reasons for missed exercise: Fatigue (*n* = 20), comorbid medical issues (*n* = 20), and competing priorities (*n* = 15)	Adverse events related to intervention: Four cases of back pain, including two mild cases (grade 1), one moderate case (grade 2), one moderate-to-severe case (grade 2–3) Adverse events unrelated to intervention: One moderate-to-severe spinal fracture (grade 2–3), one moderate back pain (grade 2) from an outdoor slip, one case of grade 2 arrhythmia in a participant with a history of cardiac intervention	Adherence was tracked directly in HEAL-Me. Adherence: 82.9%–89.9% Programme completion: Programme and fitness testing: 92.9% Follow-up questionnaires: 96.4%	Exercise programme was beneficial and enjoyable: 96.3% agreed or strongly agreed Service provided by programme staff: 92.6% excellent HEAL-Me app was burdensome: 88.9% disagree or strongly disagree 48% of participants felt the programme helped them manage cancer-related symptoms and side effects, 48% felt neutral about the programme’s benefits for symptoms and side effects Recommendation: 85.2% strongly agreed they would recommend the exercise programme to others
Schmitz et al. (2021)	Days logged in: 40.3/90	Four patients did not continue using the tablet for at least 1 month, with baseline assessments indicating significantly higher pain scores (*p* < 0.05). No other adverse symptoms or events directly associated with the intervention were reported	76% still using after 1 month	Patient satisfaction ratings were collected on 662 intervention days, walking: rated as helpful on 83% of days it was offered Psychological interventions (e.g., CBT instruction, reframing symptoms): 49% helpful; 51% not helpful Satisfaction was mixed regarding psychological interventions, with half the participants finding them helpful
Schmitz et al. (2023)	64.9% logged in at least 30 over 90-day study	No adverse events reported	Technology use after the weekly calls ended: 4 months: 58% 5 months: 47% 6 months: 29%	Average satisfaction (Client Satisfaction Questionnaire): 25.36 (scale 8–32, 32 best) CEQ-Scale Subscales: Logical: 7.42/10 Help with symptoms: 5.76/10 Recommend to friend: 7.24/10 Improvement in symptoms: 47.81% (scale 0–100%) System Usability Survey mean 86.14 (scale 0–100, 100 best) General satisfaction: 70% or higher across all interventions
Shachar et al. (2023)	Twenty-four study participants (46%) had analysable Fitbit data at 3 months follow-up	During active treatment: Participants had 1 (12%) or 2 (8%) hospitalisations	40 (77% of 52) completed the 3-month 29 (73% of 40) completed the 6-month follow-up	NM
Soh et al. (2018)	App group: 176/203 (86.7%) completed the programme Health education group: content viewed 2338 times by the gastric cancer group and 3071 by the colon cancer group	NM	Overall programme completion rate: 86.7% Gastric cancer: 84.2% (85/101) completed the study Colon cancer: 89.2% (91/102) completed the study Reasons for dropout: Physical condition change: 50.0% of dropouts in gastric cancer and 54.5% in colon cancer participants Difficulty using the app: 31.3% of gastric cancer dropouts and 36.4% of colon cancer dropouts Transfer to other hospital: 12.5% for gastric cancer and 9.1% for colon cancer	Satisfaction (5 = very good, 1 = very bad), M (SD) Gastric cancer: 3.93 (0.88) Colon cancer: 4.01 (0.87)
Wang et al. (2021)	Average PA prescription in weeks 1–3: 47.0 min/week Average PA engaged at 6 weeks: 70.4 min/week	No adverse events reported	Adherence rate (first 3 weeks): 160% Adherence rate (second 3 weeks) 82% Attrition rate: 60% (6/10) Two died, three withdrew for personal reasons, one had severe depression	Satisfaction (1–4 Likert scale): mean 3.7

*PESS* Perceived Environmental Supportiveness Scale, “Above average users”, app activity for more than 9 weeks, “Below average users”, app activity for less than 9 weeks, *6MWT* 6-min walk test, *UEQ-S*, User Experience Questionnaire—Short version, *ECOG* Eastern Cooperative Oncology Group (performance status), *SpO* peripheral capillary oxygen saturation, *AT* aerobic training, *RT* resistance training, *CSQ-8* Client Satisfaction Questionnaire—8 items, *SUS* System Usability Scale, *PA* physical activity, *AEs* adverse events, *EORTC QLQ-C30* European Organisation for Research and Treatment of Cancer Quality of Life Questionnaire Core 30, *CBT* cognitive behavioural therapy, *NM* not measured

#### Efficacy outcomes

Interventions assessed efficacy by QoL, PA or function, fatigue, pain, anxiety and/or depression, dyspnoea, sleep, and nutrition. Overall, the findings were mixed. Full details for all domains are provided in Table 5 and Supplement Four.

**Table 5:** Summarised efficacy outcomes

Source	Quality of life	Physical activity/function	Fatigue and/or pain	Anxiety and/or depression	Dyspnoea, sleep, and/or nutrition
Asensio-Cuesta et al. (2024)	**EORTC QLQ-C30** Patient 1 anecdotal ↑ Patient 2 anecdotal ↑	**Steps per week** Patient 1 ↑ Patient 2 ↓ initially, then ≠ Non-significant	**EORTC fatigue and pain** Patient 1 objective ↑, subjective ≠ Patient 2 – objective ↑, subjective ≠	NM	**Dyspnoea** Patient 1 ↑ Patient 2 ≠
Bade et al. (2018)	NM	**Steps per day/week** Calls group ↑ Texts group ↑ Non-significant	NM	NM	NM
Bade et al. (2021)	**EORTC QLQ-C30** Intervention ↑ Control ↑ Between group *p* = 0.668	**MVPA (min/week)** Intervention: ↑ Control: ↑ Between group *p* = 0.051 favouring intervention **EORTC role functioning** Intervention: ↑ Control: ↓ Between group *p* = 0.022 **EORTC role functioning** Intervention: ↑ Control: ↑ Between group *p* = 0.853	**EORTC fatigue** Intervention ↑ Control ↓ Between group *p* = 0.456 **EORTC pain** Intervention ↓ Control ↓ Between group *p* = 0.948	**PHQ-9 depression** Intervention ↑ Control ↓ Between group *p* = 0.203	**EORTC Dyspnoea** Intervention ↑ Control ↓ Between group *p* = 0.051 **MMRC Dyspnoea** Intervention ↑ Control ↑ Between group *p* = 0.889 **Appetite** Intervention ↑ Control ↓ Between group *p* = 0.290 **Insomnia** Intervention ↓ Control ↑ Between group *p* = 0.643
Bergerot et al. (2025)	**FACT-G** ↑ from baseline *p* = 0.001	NM	**ESAS pain** ↑ from baseline *p* = 0.001 **ESAS fatigue** ↑ from baseline *p* = 0.001	**ESAS depression** ↑ from baseline *p* = 0.001 **ESAS anxiety** ↑ from baseline *p* = 0.001	**ESAS drowsiness** ↑ from baseline *p* = 0.001 **ESAS appetite** ↑ from baseline *p* = 0.001 **ESAS nausea** ↑ from baseline *p* = 0.001 **ESAS Dyspnoea** ≠ from baseline *p* = 0.78
Cheong et al. (2018)	**EORTC Global Health** ≠ from baseline *p* = 0.271	**Chair stand test** ↑ from baseline *p* = 0.001 **2-min walk test** ↑ from baseline *p* = 0.001 **Handgrip strength** ≠ from baseline *p* = 0.287 **IPAQ** ≠ from baseline *p* = 0.118 **EORTC physical functioning** ≠ from baseline *p* = 0.695	**EORTC fatigue** ↑ from baseline *p* = 0.007 **EORTC pain** ↑ from baseline *p* = 0.471	NM	**EORTC Dyspnoea** ≠ from baseline *p* = 0.838 **EORTC insomnia** ↑ from baseline *p* = 0.321 **EORTC appetite loss** ↑ from baseline *p* = 0.085
Cheville et al. (2012)	**FACT-G** Intervention ↑ Control ≠ Between group *p* = 0.54	**AM-PAC mobility SF** Intervention ↑ Control ≠ Between group *p* = 0.002 **AM-PAC activity SF** Intervention ↑ Control ↑ Between group *p* = 0.74	**FACT-F** Intervention ↑ Control ↓ Between group *p* = 0.03 **Pain** Intervention ↑ Control ↑ Between group *p* = 0.87	NM	**Sleep quality** Intervention ↑ Control ≠ Between group *p* = 0.002
Cheville et al. (2019)	**EQ-5D-3L** Change compared to Arm 1 Arm 2 ↑ *p* = 0.001 Arm 3 ↑ *p* = 0.08	**AM-PAC-CAT** Change compared to Arm 1 Arm 2 ↑* p* = 0.03 Arm 3 ≠ *p* = 0.41	**Pain interference** Change compared to Arm 1 Arm 2 ↑ *p* = 0.03 Arm 3 ≠ *p* = 0.41 **Pain intensity** Change compared to Arm 1 Arm 2 ↑ *p* = 0.03 Arm 3 ≠ *p* = 0.41	NM	NM
Coats et al. (2020)	**EORTC QLQ-C30** No significant changes reported	**6-min walk test** ↑ from baseline *p* = 0.01 **Timed sit-to-stand** ↑ from baseline *p* = 0.05 **TUG** ≠ from baseline *p* = 0.49 **VO₂peak** ≠ from baseline *p* = 0.74 **Strength** ≠ from baseline *p* = 0.98	NM	NM	NM
Crosby et al. (2023)	**EORTC global health** ↑ from baseline *p* = 0.888	**IPAQ-SF** ↑ from baseline *p* = 0.047 **Two-min step test** ↑ from baseline *p* < 0.001 **Push-up test** ↑ from baseline *p* = 0.010 **Chair rise test** ↑ from baseline *p* = 0.006 **Static balance** ↑ from baseline *p* = 0.007 **EORTC physical functioning** ↑ from baseline *p* = 0.315 **ABC scale** ↑ from baseline *p* = 0.192	**EORTC fatigue** ↑ from baseline *p* = 0.440 **EORTC pain** ↑ from baseline *p* = 0.786	NM	**EORTC Dyspnoea** ↑ from baseline *p* = 0.084 **EORTC insomnia** ≠ from baseline *p* = 1.00 **EORTC appetite loss** ≠ from baseline *p* = 0.157
Delrieu et al. (2020)	**EORTC global health** ↑ from baseline *p* = 0.74	**6-min walk test** ↑ from baseline *p* < 0.001 **Quadriceps strength** ↑ from baseline *p* < 0.001 **EORTC physical functioning** ↑ from baseline *p* = 0.17 **VO₂peak** ≠ from baseline *p* = 0.71	**EORTC fatigue** ↑ from baseline p =.08 **Piper scale fatigue** ≠ from baseline p > 0.99 **EORTC pain** ↑ from baseline *p* = 0.29	NM	**EORTC appetite loss** ↑ from baseline *p* = 0.02 **EORTC Dyspnoea** ↑ from baseline *p* = 0.70 **EORTC insomnia** ↑ from baseline *p* = 0.37
Dorion et al. (2017)	**EORTC** ≠ from baseline	**Step count** ↓ from baseline *p* = 0.02	**SF-BPI pain** ↑ from baseline	NM	NM
Evans et al. (2021)	**EORTC global health** Intervention ↑ Control ↓ Between group *p* = 0.24	**MVPA mins/day** Intervention ↑ Control ↓ Between group *p* = 0.01 **EORTC physical functioning** Intervention ↑ Control ↓ Between group *p* = 0.44	**EORTC fatigue** Intervention ↓ Control ↓ Between group *p* = 0.56 **EORTC pain** Intervention ↓ Control ↓ Between group *p* = 0.81	**HADS depression** Intervention ↑ Control ↓ Between group *p* = 0.06 **HADS anxiety** Intervention ↓ Control ↓ Between group *p* = 0.74	**EORTC Dyspnoea** Intervention ↓ Control ↓ Between group *p* = 0.40 **EORTC appetite loss** Intervention ↓ Control ↓ Between group *p* = 0.18 **EORTC insomnia** Intervention ↑ Control ↓ Between group *p* = 0.27 **PSQI sleep quality** Intervention ↓ Control ↓ Between group *p* = 0.10
Hacker et al. (2020)	**EORTC** Intervention ↑ Control ↓ Between group *p* > 0.05	**PA count per min** Intervention ↓ Control ↓ Between group *p* > 0.05 **Timed stair climb** Intervention ↓ Control ↓ Between group *p* > 0.05 **Timed up and go** Intervention ↓ Control ↓ Between group *p* > 0.05 **Sit to stand** Intervention ↑ Control ↓ Between group *p* > 0.05 **Handgrip right** Intervention ↓ Control ↓ Between group *p* > 0.05 **Handgrip left** Intervention ↓ Control ↓ Between group *p* > 0.05	**Chalder fatigue** Intervention ≠ Control ↓ Between group *p* > 0.05 **EORTC pain** Intervention ↑ Control ↓ Between group *p* > 0.05	**PROMIS anxiety** Intervention ↑ Control ↑ Between group *p* > 0.05 **PROMIS depression** Intervention ↑ Control ↑ Between group *p* > 0.05	**EORTC Dyspnoea** Intervention ↓ Control ≠ Between group *p* > 0.05 **EORTC sleep disturbance** Intervention ↑ Control ↑ Between group *p* > 0.05 **EORTC appetite loss** Intervention ↓ Control ≠ Between group *p* = 0.054
Keum et al. (2021)	**EORTC QLQ** Intervention ≠ Control ≠ Between group *p* > 0.05 **EORTC GHS** Intervention ↑ Control ↓ Between group *p* = 0.004	**Skeletal Muscle Index** Intervention ↓ Control ↓ Between group *p* = 0.011	NM	NM	**PG-SGA nutritional status** Intervention ↑ Control ↑ Between group *p* > 0.05
Kim et al. (2018)	**WHO-BREF QoL** Intervention ↓ Control ↓ Between group favouring intervention *p* = 0.01	**Physical health** Intervention ↑ Control ↑ Between group favouring control *p* = 0.003	NM	**Beck’s Depression Index** Intervention ↓ Control ↓ Between group *p* = 0.99 **State Anxiety Scale** Intervention ↓ Control ↓ Between group *p* = 0.21	NM
Lee et al. (2024)	**PROMIS global health physical** ↑ from baseline *p* = 0.020 **PROMIS global health mental** ↑ from baseline *p* = 0.113	**Godin activity score** ↑ from baseline *p* = 0.001 **Sit-to-stand** ↑ from baseline *p* = 0.046 **Grip strength right** ↑ from baseline *p* = 0.013 **Grip strength left** ↑ from baseline *p* = 0.003 **6MWT** ↑ from baseline *p* = 0.271 **Sit and reach** ↓ from baseline *p* = 0.359 **8-foot up and go** ≠ from baseline *p* = 0.846	NM	NM	**Healthy Eating Index** ↑ from baseline *p* = 0.043
Longacre et al. (2020)	**EQ-5D-3L QALYs** Intervention ↑ Control ↑ Significance NR	NM	NM	NM	NM
Low et al. (2023)	**FACT** Intervention ↓ Control ↓ Between group *p* = 0.30	**Steps per day** Intervention ↓ Control ↓ Between group *p* = 0.15 **MDASI physical symptoms** Intervention ↓ Control ↓ Between group *p* = 0.76	NM	**CES depression** Intervention ↓ Control ↓ Between group *p* = 0.22	NM
Park et al. (2019)	**Global health status/QoL** ↑ from baseline p = 0.06	**EORTC physical functioning** ↑ from baseline *p* = 0.06 **6-min walk distance** ↑ from baseline *p* = 0.001	**EORTC fatigue** ↑ from baseline *p* < 0.001 **EORTC pain** ≠ from baseline *p* = 0.33	**GAD anxiety** ↑ from baseline *p* < 0.001 **PHQ depression** ↑ from baseline *p* = 0.02	**EORTC Dyspnoea** ≠ from baseline *p* = 0.56 **EORTC insomnia** ↑ from baseline *p* = 0.12 **EORTC appetite loss** ↑ from baseline *p* = 0.047
Phillips et al. (2024)	**FACT-G** Intervention ↑ Control ↓ Between group *p* = 0.25	**PROMIS physical function** Intervention ≠ Control ↑ Between group *p* = 0.44 **FACT-B FWB** Intervention ↑ Control ↑ Between group *p* = 0.85 **SPPB** Intervention ↓ Control ↓ Between group *p* = 0.06	**PROMIS fatigue** Intervention ↑ Control ↓ Between group *p* = 0.08 **PROMIS pain interference** Intervention ↓ Control ↑ Between group *p* = 0.07	**PROMIS anxiety** Intervention ↓ Control ↓ Between group *p* = 0.89 **PROMIS depression** Intervention ≠ Control ≠ Between group *p* = 0.93	**PROMIS sleep disturbance** Intervention ↑ Control ↑ Between group *p* = 0.80 **PROMIS sleep impairment** Intervention ↑ Control ↑ Between group *p* = 0.50
Purdy et al. (2022)	**FACT-MM** ↑ from baseline ES = 0.43	**2-min step test** ↑ from baseline ES = 1.28 **Sit-to-stand** ↑ from baseline ES = 1.00 **Sit-and-reach** ↑ from baseline ES = 0.81 **FACT PWB** ≠ from baseline ES = 0.16 **FACT FWB** ≠ from baseline ES = 0.21	**FACT-fatigue** ↑ from baseline ES = 0.21	**FACT-emotional role** ↑ from baseline ES = 0.57	NM
Schmitz et al. (2021)	NM	NM	**Global fatigue** Intervention ≠ Control ↓ Between group *p* > 0.05 **Pain severity and interference** Intervention ↑ Control ↑ Between group *p* > 0.05	**Distress** Intervention ↑ Control ↓ Between group difference possible	**PSQI sleep quality** Intervention ↓ Control ↓ Between group *p* > 0.05
Schmitz et al. (2023)	**SF-36 general health** Intervention ≠ Control ≠ Between group immediate *p* = 0.76 and delayed 0.50	**Chair stands** Intervention ↑ Control ↑ Between group immediate p = 0.57 and delayed *p* = 0.30 **SF-36 physical functioning** Intervention ↑ Control ≠ Between group immediate *p* = 0.12 and delayed *p* = 0.79	**SF-36 pain** Intervention ↑ Control ↓ Between group immediate p = 0.37 and delayed *p* = 0.56 **SF-36 fatigue** Intervention ↑ Control ≠ Between group immediate *p* = 0.35 and delayed *p* = 1.00	NM	**Sleep disturbance** Intervention ≠ Control ≠ Between group immediate *p* = 0.33 and delayed *p* = 0.41
Shachar et al. (2023)	**PROMIS global health** ↑ from baseline *p* = 0.49 **FACT-G** ↑ from baseline *p* = 0.25	**PROMIS physical function** ≠ from baseline *p* = 0.97 **FACT PWB** ≠ from baseline *p* = 0.36 **FACT FWB** ↑ from baseline *p* = 0.11 **PSEFSM** ≠ from baseline *p* = 0.97 **PA minutes/week** ↑ from baseline *p* = 0.04	**PROMIS fatigue** ≠ from baseline *p* = 0.60 **PROMIS pain interference** ≠ from baseline *p* = 0.63 **FACIT-fatigue** ≠ from baseline *p* = 0.31	**PROMIS anxiety** ↑ from baseline *p* = 0.02 **PROMIS depression** ↑ from baseline *p* = 0.09 **Mental health** ≠ from baseline *p* = 0.78	**PROMIS sleep quality** ≠ from baseline p = 0.18
Wallace et al. (2025)	**PROMIS QoL** Intervention ↑ Control ↑ Between group *p* = 0.84	NM	NM	NM	NM
Wang et al. (2021)	**FACT-G** ≠ from baseline *d* = 0.0 **Lawton IADL** ≠ from baseline *d* = 0.1 **MDASI** ↓ from baseline *d* = 0.2	**Preferred gait speed** ↑ from baseline d = 0.8 **6-min walk test** ↑ from baseline *d* = 0.6 **Grip strength** ≠ from baseline *d* = 0.0	**BFI** ↓ from baseline *d* = 0.1 **Pain** ↑ from baseline *d* = 0.1	NM	NM
Wolf et al. (2024)	Not reported	NM	NM	**PHQ-9** ↑ from baseline *p* = 0.12	NM

*NM* not measured, *EORTC QLQ-C30* European Organisation for Research and Treatment of Cancer Quality of Life Questionnaire-Core 30, *FACT-G* functional assessment of cancer therapy – general, *FACT-F* functional assessment of cancer therapy – fatigue, *FACT PWB* functional assessment of cancer therapy – physical well-being, *FACT FWB* functional assessment of cancer therapy – functional well-being, *FACT-MM* functional assessment of cancer therapy – multiple myeloma, *FACT-B* functional assessment of cancer therapy – breast, *FACT-Emotional role* functional assessment of cancer therapy – emotional well-being, *FACIT-Fatigue* functional assessment of chronic illness therapy – fatigue, *PHQ-9* Patient Health Questionnaire-9, *PROMIS* patient-reported outcomes measurement information system, *SF-36* Short Form-36 Health Survey, *SF-BPI* Short Form Brief Pain Inventory, *ESAS* Edmonton Symptom Assessment System, *HADS* Hospital Anxiety and Depression Scale, *GAD* Generalised Anxiety Disorder Scale, *PG-SGA* Patient-Generated Subjective Global Assessment, *MDASI* MD Anderson Symptom Inventory, *BFI *Brief Fatigue Inventory, *PSQI* Pittsburgh Sleep Quality Index, *ABC Scale* Activities-specific Balance Confidence Scale, *IPAQ* International Physical Activity Questionnaire, *IPAQ-SF* International Physical Activity Questionnaire – Short Form, *MVPA* Moderate-to-Vigorous Physical Activity, *6MWT* 6-min walk test, *TUG* timed up and go test, *SPPB* short physical performance battery, *AM-PAC* activity measure for post-acute care, *AM-PAC-CAT* AM-PAC computer adaptive test, *EQ-5D-3L* EuroQol 5-dimension 3-level instrument, *QALYs* quality-adjusted life years, *PSEFSM* physical self-efficacy functional subscale measure, *MMRC Dyspnoea* Modified Medical Research Council Dyspnoea Scale, *Lawton IADL* Lawton Instrumental Activities of Daily Living Scale

#### Quality of life/health status

Quality of life was assessed primarily using the EORTC QLQ-C30, FACT-G, and EQ-5D-3L with some using tools such as MD Anderson Symptom Inventory, Lawton Instrumental Activity of Daily Living Scale, FACT-MM, SF-36 General Health, PROMIS Global Health, and the PHQ-9.

Digital interventions had limited overall impact on QoL and health status. Of the 29 studies, four did not assess QoL outcomes [32, 42, 49, 50]. Among the remaining 25 studies, eight (32%) reported significant improvements in at least one QoL or global health status measure. These included gains in global health status, physical health scores, or quality-adjusted life years, with *p*-values ranging from 0.001 to 0.02, and one study reporting a moderate effect size (e.g., ES = 0.43) [26, 33–36, 43, 44, 52].

In contrast, 17 studies (68%) found no significant change in QoL throughout the interventions, with QoL typically remaining stable [25, 27–31, 37–42, 45–48, 51].

#### Physical activity/function

Of the 29 studies, 27 (93%) assessed PA and/or functional capacity outcomes. Among these, ten studies (37%) demonstrated significant improvements in daily step counts, walking distance (e.g., 6-min walk test), moderate-to-vigorous PA, or mobility. Reported *p*-values ranged from 0.001 to 0.03, and one study demonstrated significance via 95% confidence intervals [26–30, 33, 37, 48, 51]. One study also reported moderate-to-large effect sizes (e.g., ES = 1.28) [52].

Six studies assessed functional strength using measures such as chair stand tests, grip strength, push-up tests, or isometric muscle strength. Of these, five studies reported significant improvements (*p*-values ranging from < 0.001 to 0.046, or via confidence intervals not crossing zero). These included meaningful gains in lower and upper body strength and endurance [28, 29, 31, 44, 51, 52].

One study assessed the impact of chemotherapy on the Skeletal Muscle Index (SMI) [33]. While both Noom users and non-users experienced declines in SMI during treatment, the decrease was substantially smaller among Noom users (− 3.27%) compared to non-users (− 13.96%). However, this between-group difference did not reach statistical significance (*p* = 0.11).

#### Fatigue and pain

Eighteen studies assessed fatigue (62%) [24, 25, 27–32, 37–40, 43, 45, 48, 49, 51, 52]. Of these, four studies (22%) reported significant changes (*p* < 0.001 − *p* = 0.03) in fatigue [37, 43, 48, 51]. The remaining 14 studies reported either no significant change or only minimal reductions in fatigue levels.

Nine studies assessed pain (66%) [24–32, 37–41, 43, 45, 48, 49, 51]. Significant reductions in pain were reported in three studies (17%) [26, 38, 43]. Cheville et al. (2019) reported improvements in both pain interference and intensity (*p* = 0.01–0.006), and Bergerot et al. (2025) found significant reductions in pain levels from baseline to follow-up (*p* = 0.001). Schmitz et al. (2023) reported mixed findings: while no significant effects were seen using the SF-36 pain subscale, the Brief Pain Inventory (BPI) showed significant improvements in pain severity and interference in the delayed treatment group compared to controls (*p* = 0.020–0.033). The remaining 15 studies (78.95%) reported no significant changes in pain.

Schmitz et al. (2023) used two different pain measures and found a non-significant change in pain at 3 months between the immediate treatment group and control group with the SF-36 Pain subscale. However, using the BPI, the delayed treatment group vs the control group experienced a 6-month decrease in pain severity and a significant reduction in pain interference [38].

#### Anxiety and depression

Anxiety [27, 30, 31, 34, 37, 39, 43, 45] and depression [25, 27, 30, 31, 34, 36, 37, 39, 43, 45] were assessed in 8/10 studies, respectively. Results were mixed, with six studies showing small, statistically nonsignificant reductions in anxiety and depression scores [25, 27, 31, 34, 39, 45].

Three studies reported statistically significant improvements in depressive symptoms. Park et al. (2019) observed a reduction in depression scores at 12 weeks (*p* = 0.02), while Bergerot et al. (2025) reported a substantial post-intervention decrease in depressive symptoms (*p* = 0.001). In contrast, Low et al. (2023) reported depressive symptoms worsened post-surgery, with no between-group differences, highlighting the potential influence of clinical context.

Similarly, three studies demonstrated significant improvements in anxiety symptoms. Park et al. (2019) found significant reductions at 12 weeks (*p* < 0.001), Shachar et al. (2023) reported decreased PROMIS anxiety scores over three months (*p* = 0.02), and Bergerot et al. (2025) observed a significant reduction in anxiety symptoms from baseline to post-intervention (*p* = 0.001).

#### Dyspnoea,sleep, and nutrition

Dyspnoea outcomes were reported in 9/29 included studies (31%) [24, 25, 28–31, 37, 43, 51], most using the EORTC QLQ-C30 dyspnoea subscale [24, 25, 28–31, 37, 51], while one study incorporated the MMRC scale [25] and another used the Edmonton Symptom Assessment System (ESAS) [43].

Findings were inconsistent across studies, with seven (77.78%) reporting minimal or non-significant changes in dyspnoea symptoms [24, 25, 29, 31, 37, 43, 51], and two studies (22.22%) reported non-significant worsening dyspnoea symptoms [28, 30].

Sleep-related outcomes were reported in 13/29 studies (45%) using the EORTC [25, 28–31, 37, 51] sleep symptom scale, the Pittsburgh Sleep Quality Index [30, 49], an 11-point scale [48], the Patient Reported Outcomes Measurement Information System (PROMIS) [31, 39, 45], ESAS [43], and a Sleep Disturbance scale [31, 38]. Two studies (15%) illustrated significant improvement in sleep outcomes. Bergerot et al. (2025) found a significant reduction in drowsiness (mean decrease from 1.8 to 0.4; *p* = 0.001), and Cheville et al. (2013) reported improved sleep quality in the intervention group compared to control (mean change = 1.46 vs –0.10; *p* = 0.002). The remaining 11 studies (84%) found no statistically significant effects.

Nutritional outcomes were reported in 11/29 studies (38%), with the EORTC questionnaire [25, 28–31, 37, 51], the Patient-generated Subjective Global Index [33], the web-based Diet History Questionnaire II, the Healthy Eating Index (HEI, 2015) [44], ESAS [43], and adverse nutritional event reporting [34]. Four studies reported significant changes (4/11; 36%). Delrieu et al. (2020) found a significant reduction in appetite loss on the EORTC (mean change =  − 10.9; *p* = 0.02), and Bergerot et al. (2025) reported a reduction in appetite-related symptom scores on the ESAS (mean decrease from 2.2 to 0.6; *p* = 0.001). Keum et al. (2021) found significantly lower protein and energy intake among Noom users and below-average users compared to above-average users at 12 weeks (e.g. protein intake *p* = 0.02; energy intake *p* = 0.04). Lee et al. (2024) observed significant improvements in HEI scores, calorie and protein intake, and reductions in saturated fat (*p* = 0.014–0.043), although no significant changes were seen in carbohydrate or fat intake, fruit and vegetable consumption, or added sugar (*p* > 0.05).

The remaining seven studies (64%) did not report statistically significant changes in nutrition-related outcomes. These included small, non-significant improvements or stable scores on appetite or dietary measures across intervention periods. Efficacy results are summarised in Table 5 with full details in supplemental materials.

## Discussion

This systematic review examined the feasibility, acceptability, and efficacy of digital lifestyle interventions for people living with incurable cancer. Findings suggest these interventions are largely feasible and acceptable, with encouraging retention and satisfaction rates. However, evidence for efficacy was inconsistent and often limited.

Most studies demonstrated that digital interventions were feasible to deliver and acceptable to participants, with high retention and generally positive user feedback. Engagement appeared more sustained in PA interventions compared to education-only content, aligning with patterns seen in curative cancer populations, where interactivity and structure promote adherence [4]. The ability of digital platforms to overcome barriers related to mobility or geography makes them particularly well-suited for this population [53].

Importantly, older adults (≥ 65y) were represented in several studies. Although few interventions were tailored specifically for them, evidence suggests that with appropriate support, older adults can and do engage meaningfully with digital tools [54, 55]. This highlights the potential for digital platforms to be inclusive, though age-related adaptations should be built into future designs.

In contrast to feasibility and acceptability, evidence for efficacy was limited. Only a minority of studies reported significant improvements in outcomes such as sleep, physical function, appetite, or QoL. This mirrors previous findings in advanced cancer populations, where the effects of behavioural interventions are often modest and variable [4, 56]. While PA interventions showed potential for functional gains, nutritional and multimodal interventions were fewer and less conclusive.

Similarly, nutrition plays a critical and complementary role, with research indicating that poorer nutritional status, including malnutrition and sarcopenia, is associated with reduced QoL and worse clinical outcomes in cancer populations [57]. However, there is a paucity of evidence exploring digital interventions that address nutritional outcomes, particularly in combination with PA, for individuals with incurable cancer. This represents an important gap in the field and a key opportunity for future intervention design.

Measurement inconsistency also restricted comparison. Tools varied widely across studies (e.g. EORTC, PROMIS, and ESAS), with some outcomes showing conflicting results depending on the measure used, as seen in Bade et al. (2021). Standardising outcome measures could support synthesis and comparability in future trials.

Compared to the curatively treated cancer population, where structured exercise and dietary interventions have demonstrated more consistent effects on physical function and QoL [56], the evidence in incurable cancer populations remains limited. This may not reflect a true lack of efficacy, but rather a lack of adequately powered studies using standardised outcome measures in this specific cohort. Few trials reported formal sample size calculations, and heterogeneity in tools and endpoints makes comparisons challenging. Future research should prioritise well-designed, adequately powered studies that use consistent, validated outcomes to understand better the potential of these interventions in the incurable cancer setting.

Including co-designed features, such as personalised feedback, caregiver support, or social engagement tools, may improve uptake and adherence. Interventions that incorporate social interaction may also address isolation and improve mental wellbeing, both of which are critical in this population [58, 59].

Mixed-methods designs are needed to evaluate both impact and experience. Studies should report both quantitative and qualitative outcomes and engage patients in design and evaluation [60]. Particularly for patients near the end of life, balancing objective data collection with reduced burden is critical. A hybrid approach, combining digital tools with minimal in-person or telephone-based support, may offer the best compromise between rigour and practicality.

### Strengths, limitations, and future considerations

To our knowledge, this is the first systematic review to explore the current evidence and evaluate the feasibility, acceptability, and potential efficacy of digital interventions supporting healthy lifestyle behaviours to promote wellbeing and independence among adults living with advanced or incurable cancer. It followed best-practice standards (e.g. pre-registration and PRISMA adherence). The broad range of interventions and technologies included reflects the versatility of digital approaches but limits the depth of analysis for specific areas. Heterogeneity in design, outcome measures, small sample sizes, and underrepresentation of older adults also constrained synthesis and generalisability.

Several key considerations for the future can be drawn from these findings and the broader literature. Firstly, for older adults (≥ 65 years) and those nearing the end of life, hybrid or in-person data collection methods (e.g. home visits and telephone interviews) may improve data accuracy and provide a more supportive experience. Future research should also consider user-centred or co-design methodologies, allowing older adults with incurable cancer to inform the development and testing of digital tools through iterative usability testing. To ensure the “patient voice” is meaningfully captured, researchers should use structured patient and public involvement approaches, such as advisory panels, participatory workshops, and guidance from frameworks like the INVOLVE guidelines or GRIPP2 checklists. Standardising outcome measurement using validated items would allow for better cross-study comparability and understanding. Finally, mixed-methods study designs, combining quantitative outcomes with qualitative feedback, are recommended to evaluate both the impact and experience of digital interventions. These methodological considerations will help ensure future research is rigorous, relevant, and responsive to the needs of people living with incurable cancer.

## Conclusion

Digital lifestyle interventions appear feasible and acceptable for people with incurable cancer, but evidence for efficacy remains limited. To optimise future interventions, researchers should prioritise co-design or user-centred design with patients, standardise outcomes, and evaluate tailored, integrated approaches. Future trials should adopt mixed-methods designs, include underrepresented groups, and balance rigour with real-world feasibility to ensure digital tools are both effective and meaningful.

## Supplementary Information

Below is the link to the electronic supplementary material.

Supplemental One_PRISMA (DOCX 39.9 KB)

Supplemental Two; Appendix (DOCX 51.4 KB)

Supplemental Three (DOCX 57.4 KB)

Supplement Four (DOCX 72.2 KB)

## Data Availability

No datasets were generated or analysed during the current study.

## Abbreviations

PA: Physical Activity
PRISMA: Preferred Reporting Items for Systematic Reviews and Meta-Analyses
QoL: Quality of Life
RCT: Randomised Controlled Trial
MMRC: Medical Research Council
PROMIS: The Patient-Reported Outcomes Measurement Information System
EQ5-LD: EuroQol 5-Dimension 3-Level
EORTC QLQ-C30: European Organisation for Research and Treatment of Cancer Quality of Life Questionnaire
PG-SGA: Patient-generated Subjective Global Index
SMI: Skeletal Muscle Index
FACT-G: Functional Assessment of Cancer Therapy-General
FACT-MM: Functional Assessment of Cancer Therapy-Multiple Myeloma
Cochrane EPOC: Cochrane Effective Practice and Organisation of Care
SF-36: Short Form Health Survey
BPI: Brief Pain Inventory
