# Supplementary material for: Using digital technology to support wellbeing and independence among people living with incurable cancers: a systematic review
Source: Support Care Cancer. 2025 Jul 18;33(8):699. doi: 10.1007/s00520-025-09759-1 (PMC12274145; doi:10.1007/s00520-025-09759-1)
Supplement: Supplementary file 2 — Supplemental Two; Appendix (DOCX 51.4 KB) [file 520_2025_9759_MOESM2_ESM.docx]

## Appendix: search development and strategies for all databases

The search strategy was initially developed using MEDLINE All via OVID using three main concepts: incurable cancer AND digital technologies AND exercise/diet/lifestyle. Each concept was developed by examining the bibliographic details of known, relevant results to determine potential Indexed Terms and free text terms, tested against another set of relevant results and revised if necessary. The final MEDLINE search was adapted for the remaining databases and was conducted by (SG). The initial search was conducted on 1^st^ December 2021 and updated on 29^th^ November 2023 and 9^th^ May 2025. We updated the searches by re-running the saved search in each database and applying a database specific filter to limit results to those added to each database since the date of the last search. For example, an extra line was added to the Medline search for the November 2023 update search to only retrieve results from a couple of days before the initial search until the update search date using the dt (create date) field:

limit 64 to dt=20211129-20231129

We also performed forward and backward citation searching on the included studies identified from the original search via Citation Chaser on 1^st^ December 2023, importing results into Endnote and removing duplicates already retrieved from database searches and non-cancer-related citations before screening ( using the Endnote search function: NOT any field contains (cancer or neoplasm or oncology or tumour or tumor).

Full search strategies for each database including platform details and inception dates are listed below.

## Medline via OVID (1946 onwards)

1. exp NEOPLASMS/

2. (cancer* or neoplas* or tumor* or tumour* or malignan* or carcinoma* or oncolog* or leukemi* or leukaemi* or lymphoma* or myeloma* or sarcoma*).ti,ab,kw.

3. 1 or 2

4. (incurable or advanced or inoperable or unresectable or metasta* or palliat* or terminal* or "stage 4" or "stage iv" or secondary or recurren*).ti,ab,kw. or Palliative Care/

5. ((late or advanced or last or end or final) adj3 (phase* or stage*)).ti,ab,kw.

6. 4 or 5

7. (3 and 6) or Cancer Survivors/ or (cancer* adj survivor*).mp. [Advanced/incurable cancer concept]

8. wearable electronic devices/ or fitness trackers/ or smart glasses/

9. "Internet of Things"/ or "Internet of Things".ti,ab,kw.

10. Accelerometry/

11. artificial intelligence/ or machine learning/

12. telemetry/ or remote sensing technology/

13. ((smart or connected) adj3 (home* or device* or applianc* or plug* or sensor* or monitor*)).ti,ab,kw.

14. IoT.ti,ab,kw.

15. acceleromet*.ti,ab,kw.

16. ((sensor* adj3 (motion or door or smart or monitor*)) or (electronic adj2 symptom* adj2 monitor*)).ti,ab,kw.

17. ((frailty adj2 monitor*) or ((computer* or electronic or technol*) adj2 (assessment* or monitoring))).ti,ab,kw.

18. ((non-instrusive or nonintrusive or passive or unobtrusive or remote*) adj2 (sensor* or monitor*)).ti,ab,kw.

19. ambient assisted living.ti,ab,kw. or ambient intelligence.mp.

20. real-time monitoring.ti,ab,kw.

21. wearable*.ti,ab,kw.

22. (sensor* adj3 (network or wearable)).ti,ab,kw.

23. inertial sensor*.ti,ab,kw.

24. (((fitness or step* or activity) adj2 (band* or tracker* or counter* or monitor*)) or fitbit*).ti,ab,kw.

25. (smart adj2 (speaker* or watch or cloth* or bed* or ring*)).ti,ab,kw.

26. exp internet/ or internet-based intervention/ or ((online or internet) adj2 intervention*).mp.

27. exp Telemedicine/ or (telehealth or telerehabilitation or telenursing or distance-based or broad-reach or (remote* adj2 deliver*)).mp.

28. Mobile Applications/

29. electronic mail/ or videoconferencing/ or wireless technology/

30. cell phone/ or smartphone/ or text messaging/

31. (telemedicine or mobile app* or smartphone* or (digital* adj2 (health or intervention*)) or mhealth or m-health or ehealth or e-health or sms or texting or text messag* or website* or web-based or web based or wireless or wearable* or ((tablet* or handheld*) adj computer*)).mp.

32. Video Games/

33. exp user-computer interface/

34. (active adj2 gam*).mp.

35. (exergam* or nintendo wii or xbox kinect or wii fit).mp. [mp=title, abstract, original title, name of substance word, subject heading word, floating sub-heading word, keyword heading word, organism supplementary concept word, protocol supplementary concept word, rare disease supplementary concept word, unique identifier, synonyms]

36. Computers, Handheld/

37. ((digital or voice) adj assistant*).mp.

38. (google assistant or SIRI or Alexa).ti,ab,kw.

39. speech recognition software.mp.

40. Patient Portals/

41. or/8-40 [smart home/tech concept]

42. exercise/ or cool-down exercise/ or gymnastics/ or muscle stretching exercises/ or exp physical conditioning, human/ or circuit-based exercise/ or endurance training/ or high-intensity interval training/ or plyometric exercise/ or resistance training/ or preoperative exercise/ or running/ or jogging/ or marathon running/ or swimming/ or walking/ or stair climbing/ or warm-up exercise/

43. exercise therapy/ or endurance training/ or motion therapy, continuous passive/ or muscle stretching exercises/ or plyometric exercise/ or resistance training/ or exp Exercise Movement Techniques/

44. (physical activity or fitness or conditioning or exercis* or exerciz*).ti,ab,kw.

45. Sedentary Behavior/

46. sedentary.ti,ab,kw.

47. tai ji/ or yoga/

48. (tai ji or t'ai chi or tai chi or taiji or taijiquan).ti,ab,kw.

49. yoga.ti,ab,kw.

50. yogic.ti,ab,kw.

51. Qigong/

52. (qigong or qi gong or ch'i kung).ti,ab,kw.

53. ((holistic or mindful*) adj2 movement*).ti,ab,kw.

54. active lifestyle*.ti,kw.

55. exp Sports/

56. exp Diet/

57. exp nutrition therapy/ or diet therapy/

58. exp Healthy Lifestyle/

59. (diet* or nutrition* or lifestyle*).mp.

60. Diet, Healthy/

61. or/42-60 [exercise, diet, nutrition]

62. exp animals/ not humans.sh. [isolate animal studies]

63. 7 and 41 and 61 [advanced cancer AND tech AND diet/exercise]

64. 63 not 62

## Embase via OVID (1974 onwards)

1. advanced cancer/

2. exp malignant neoplasm/ or cancer patient/

3. (cancer* or neoplas* or tumor* or tumour* or malignan* or carcinoma* or oncolog* or leukemi* or leukaemi* or lymphoma* or myeloma* or sarcoma*).ti,ab,kw.

4. 2 or 3

5. exp palliative therapy/

6. (incurable or advanced or inoperable or unresectable or metasta* or palliat* or terminal* or "stage 4" or "stage iv" or secondary or recurren*).ti,ab,kw.

7. ((late or advanced or last or end or final) adj3 (phase* or stage*)).ti,ab,kw.

8. 5 or 6 or 7

9. 4 and 8

10. cancer survivor/

11. (cancer* adj survivor*).mp.

12. 1 or 9 or 10 or 11

13. 1 or 9 or 10 or 11 [Advanced/incurable cancer concept]

14. activity tracker/ or smart watch/

15. "internet of things"/

16. "Internet of Things".ti,ab,kw.

17. remote sensing/

18. accelerometer/

19. accelerometry/

20. ambient intelligence/

21. ((smart or connected) adj3 (home* or device* or applianc* or plug* or sensor* or monitor*)).ti,ab,kw.

22. ((frailty adj2 monitor*) or ((computer* or electronic or technol*) adj2 (assessment* or monitoring))).ti,ab,kw.

23. ((non-instrusive or nonintrusive or passive or unobtrusive or remote*) adj2 (sensor* or monitor*)).ti,ab,kw.

24. (ambient assisted living or ambient intelligence).ti,ab,kw.

25. real-time monitoring.ti,ab,kw.

26. wearable*.ti,ab,kw.

27. (sensor* adj3 (network or wearable)).ti,ab,kw.

28. inertial sensor/

29. inertial sensor*.ti,ab,kw.

30. wearable sensor/

31. (((fitness or step* or activity) adj2 (band* or tracker* or counter* or monitor*)) or fitbit*).ti,ab,kw.

32. (smart adj2 (speaker* or watch or cloth* or bed* or ring*)).ti,ab,kw.

33. web-based intervention/

34. ((online or internet) adj2 intervention*).mp.

35. exp telemedicine/

36. (telehealth or telerehabilitation or telenursing or distance-based or broad-reach or (remote* adj2 deliver*)).mp.

37. exp mobile application/

38. e-mail/

39. videoconferencing/

40. wireless communication/

41. smartphone/

42. text messaging/

43. (telemedicine or mobile app* or smartphone* or (digital* adj2 (health or intervention*)) or mhealth or m-health or ehealth or e-health or sms or texting or text messag* or website* or web-based or web based or wireless or wearable* or ((tablet* or handheld*) adj computer*)).mp.

44. exp video game/

45. ((active adj2 gam*) or (exergam* or nintendo wii or xbox kinect or wii fit)).mp.

46. tablet computer/

47. ((digital or voice) adj assistant*).mp.

48. (google assistant or SIRI or Alexa).ti,ab,kw.

49. automatic speech recognition/

50. patient portal*.ti,ab.

51. or/14-50 [smart home/tech concept]

52. exp exercise/

53. exp kinesiotherapy/

54. (physical activity or fitness or conditioning or exercis* or exerciz*).ti,ab,kw.

55. exp sedentary time/

56. sedentary lifestyle/

57. sedentary.ti,ab,kw.

58. exp yoga/

59. Tai Chi/

60. (tai ji or t'ai chi or tai chi or taiji or taijiquan).ti,ab,kw.

61. (yoga or yogic).ti,ab,kw.

62. qigong/

63. (qigong or qi gong or ch'i kung).ti,ab,kw.

64. ((holistic or mindful*) adj2 movement*).ti,ab,kw.

65. exp sport/

66. exp diet/

67. diet therapy/

68. (diet* or nutrition* or lifestyle*).mp.

69. healthy lifestyle/

70. or/52-69

71. 13 and 51 and 70

72. limit 71 to conference abstract status [isolate conf abstracts]

73. limit 72 to yr="2021 -Current" [isolate recent conf abstracts]

74. 71 not 72 [remove all conf abstracts]

75. 73 or 74 [add in only recent conf abstracts]

76. (animal$ not human$).sh,hw. [isolate animal only studies]

77. 75 not 76 [remove animal only studies]

PsycINFO via OVID (1967 onwards)

1. exp Terminal Cancer/

2. metastasis/

3. exp Neoplasms/ and exp Survivors/

4. (cancer adj2 survivor*).ti,ab.

5. (cancer* or neoplas* or tumor* or tumour* or malignan* or carcinoma* or oncolog* or leukemi* or leukaemi* or lymphoma* or myeloma* or sarcoma*).ti,ab.

6. exp Neoplasms/

7. 5 or 6

8. (incurable or advanced or inoperable or unresectable or metasta* or palliat* or terminal* or "stage 4" or "stage iv" or secondary or recurren*).ti,ab.

9. exp Palliative Care/

10. ((late or advanced or last or end or final) adj3 (phase* or stage*)).ti,ab.

11. 8 or 9 or 10

12. 7 and 11

13. 1 or 2 or 3 or 4 or 12

14. exp wearable devices/ or exp digital technology/ or exp mobile devices/ or exp mobile health/ or exp wireless technologies/

15. exp Artificial Intelligence/ or exp Human Computer Interaction/

16. exp Internet/

17. exp telemedicine/ or digital interventions/

18. mobile applications/ or mobile technology/ or digital gaming/ or mobile phones/ or smartphones/ or tablet computers/

19. exp Teleconferencing/ or exp Videoconferencing/

20. exp Computer Games/

21. exp Technology/ or exp Computer Applications/

22. ("Internet of Things" or IoT or acceleromet* or wearable*).ti,ab.

23. ((smart or connected) adj3 (home* or device* or applianc* or plug* or sensor* or monitor*)).ti,ab.

24. ((sensor* adj3 (motion or door or smart or monitor*)) or (electronic adj2 symptom* adj2 monitor*)).ti,ab.

25. ((frailty adj2 monitor*) or ((computer* or electronic or technol*) adj2 (assessment* or monitoring))).ti,ab.

26. ((non-instrusive or nonintrusive or passive or unobtrusive or remote*) adj2 (sensor* or monitor*)).ti,ab.

27. ambient assisted living.ti,ab.

28. ambient intelligence.ti,ab.

29. real-time monitoring.ti,ab.

30. (sensor* adj3 (network or wearable)).ti,ab.

31. inertial sensor*.ti,ab.

32. (((fitness or step* or activity) adj2 (band* or tracker* or counter* or monitor*)) or fitbit*).ti,ab.

33. (smart adj2 (speaker* or watch or cloth* or bed* or ring*)).ti,ab.

34. (telehealth or telerehabilitation or telenursing or distance-based or broad-reach or (remote* adj2 deliver*)).ti,ab.

35. (telemedicine or mobile app* or smartphone* or (digital* adj2 (health or intervention*)) or mhealth or m-health or ehealth or e-health or sms or texting or text messag* or website* or web-based or web based or wireless or wearable* or ((tablet* or handheld*) adj computer*)).mp.

36. (active adj2 gam*).mp.

37. (exergam* or nintendo wii or xbox kinect or wii fit).mpmp.

38. ((digital or voice) adj assistant*).mp.

39. (google assistant or SIRI or Alexa).ti,ab.

40. exp Automated Speech Recognition/

41. or/14-40

42. exercise/ or physical activity/ or aerobic exercise/ or weightlifting/ or yoga/ or kinesiology/ or movement therapy/ or physical fitness/

43. (physical activity or fitness or conditioning or exercis* or exerciz*).ti,ab.

44. exp Activity Level/ or exp Sedentary Behavior/

45. sedentary.ti,ab.

46. yoga/

47. (yoga or yogic).ti,ab.

48. (tai ji or t'ai chi or tai chi or taiji or taijiquan).ti,ab.

49. (qigong or qi gong or ch'i kung).ti,ab.

50. ((holistic or mindful*) adj2 movement*).ti,ab.

51. exp Sports/

52. exp Diets/

53. (diet* or nutrition* or lifestyle*).mp.

54. or/42-53

55. 13 and 41 and 54

## CINAHL via Ebscohost (1937 onwards)

| S1 | (MH "Neoplasms+") |
| --- | --- |
| S2 | TI ( cancer* or neoplas* or tumor* or tumour* or malignan* or carcinoma* or oncolog* or leukemi* or leukaemi* or lymphoma* or myeloma* or sarcoma* ) OR AB ( cancer* or neoplas* or tumor* or tumour* or malignan* or carcinoma* or oncolog* or leukemi* or leukaemi* or lymphoma* or myeloma* or sarcoma* ) |
| S3 | S1 OR S2 |
| S4 | TI ( incurable or advanced or inoperable or unresectable or metasta* or palliat* or terminal* or "stage 4" or "stage iv" or secondary or recurren* ) OR AB ( incurable or advanced or inoperable or unresectable or metasta* or palliat* or terminal* or "stage 4" or "stage iv" or secondary or recurren* ) |
| S5 | (MH "Palliative Care") OR (MH "Palliative Medicine") OR (MH "Terminal Care") |
| S6 | TI ( ((late or advanced or last or end or final) N3 (phase* or stage*)) ) OR AB ( ((late or advanced or last or end or final) N3 (phase* or stage*)) ) |
| S7 | S4 or S5 or S6 |
| S8 | (MH "Cancer Survivors") OR TI cancer* N1 survivor* OR AB cancer* N1 survivor* |
| S9 | S3 AND S7 |
| S10 | S8 OR S9 |
| S11 | TI ( (smart n2 (speaker* or watch or cloth* or bed* or ring*)) ) OR AB ( (smart n2 (speaker* or watch or cloth* or bed* or ring*)) ) |
| S12 | TI ( ((fitness n2 (band* or tracker*)) or fitbit*) ) OR AB ( ((fitness n2 (band* or tracker*)) or fitbit*) ) |
| S13 | MW wearable* OR TI wearable* |
| S14 | TI ( ((non-instrusive or nonintrusive or passive or unobtrusive or remote*) n2 (home* or sensor* or monitor*)). ) OR AB ( ((non-instrusive or nonintrusive or passive or unobtrusive or remote*) n2 (home* or sensor* or monitor*) ) |
| S15 | TI ( (sensor* n3 monitor*) or electronic symptom monitor* ) OR AB ( ((sensor* n3 monitor*) or electronic symptom monitor* ) |
| S16 | TI ( ((smart or connected) n3 (home* or device* or applianc* or plug* or sensor* or monitor*)) ) OR AB ( ((smart or connected) n3 (home* or device* or applianc* or plug* or sensor* or monitor*)) ) |
| S17 | (MH "Internet of Things") |
| S18 | (MH "Wearable Sensors+") |
| S19 | TX ambient assisted living |
| S20 | (MH "Assistive Technology") |
| S21 | (MH "Telehealth+") |
| S22 | TI ( telehealth or telerehabilitation or telenursing or distance-based or broad-reach ) OR AB ( telehealth or telerehabilitation or telenursing or distance-based or broad-reach ) |
| S23 | TI remote* n1 deliver* OR AB remote* n1 deliver* |
| S24 | (MH "Mobile Applications") |
| S25 | TI ( mobile app* or smartphone* or mhealth or m-health or ehealth or digital health or digital intervention* or e-health or sms or texting or text messag* or website* or web-based or web based or wireless ) OR AB ( mobile app* or smartphone* or mhealth or m-health or digital health or digital intervention* or ehealth or e-health or sms or texting or text messag* or website* or web-based or web based or wireless ) |
| S26 | (MH "Video Games") |
| S27 | TI active N2 gam* OR AB active N2 gam* |
| S28 | TI ( exergames or exergaming or active video games or nintendo wii or xbox kinect or wii fit ) OR AB ( exergames or exergaming or active video games or nintendo wii or xbox kinect or wii fit ) |
| S29 | (MH "Computers, Hand-Held") OR (MH "Computers, Portable") |
| S30 | TI ( ((digital or voice) N1 assistant*) ) OR AB ( ((digital or voice) N1 assistant*) ) |
| S31 | AB (google assistant or SIRI or Alexa) |
| S32 | TI (google assistant or SIRI or Alexa) |
| S33 | (MH "Voice Recognition Systems") |
| S34 | S11 OR S12 OR S13 OR S14 OR S15 OR S16 OR S17 OR S18 OR S19 OR S20 OR S21 OR S22 OR S23 OR S24 OR S25 OR S26 OR S27 OR S28 OR S29 OR S30 OR S31 OR S32 OR S33 |
| S35 | (MH "Exercise+") OR (MH "Endurance Training") OR (MH "High-Intensity Interval Training") OR (MH "Warm-Up Exercise") OR (MH "Postactivation Potentiation") OR (MH "Exercise Intensity") OR (MH "Abdominal Exercises") OR (MH "Aerobic Exercises") OR (MH "Anaerobic Exercises") OR (MH "Callisthenics") OR (MH "Back Exercises") OR (MH "Group Exercise") OR (MH "Lower Extremity Exercises") OR (MH "Muscle Strengthening") OR (MH "Pilates") OR (MH "Plyometrics") OR (MH "Stretching") OR (MH "Upper Extremity Exercises") OR (MH "Walking") |
| S36 | (MH "Therapeutic Exercise+") |
| S37 | TI ( physical activity or fitness or conditioning or exercis* or exerciz* ) OR AB ( physical activity or fitness or conditioning or exercis* or exerciz* ) |
| S38 | (MH "Life Style, Sedentary+") |
| S39 | TI sedentary OR AB sedentary |
| S40 | (MH "Sports+") |
| S41 | TI ( tai ji or t'ai chi or tai chi or taiji or taijiquan ) OR AB ( tai ji or t'ai chi or tai chi or taiji or taijiquan ) |
| S42 | TI ( yoga or yogic ) OR AB ( yoga or yogic ) |
| S43 | (MH "Tai Chi") OR (MH "Yoga+") OR (MH "Dance Therapy") |
| S44 | TI ( (holistic or mindful*) N1 movement* ) OR AB ( (holistic or mindful*) N1 movement* ) |
| S45 | (MH "Diet+") |
| S46 | (MH "Nutrition+") |
| S47 | TI ( diet* or nutrition* or lifestyle* ) OR AB ( diet* or nutrition* or lifestyle* ) |
| S48 | S35 OR S36 OR S37 OR S38 OR S39 OR S40 OR S41 OR S42 OR S43 OR S44 OR S45 OR S46 OR S47 |
| S49 | S10 AND S34 AND S48 |

## Web of Science Core Collection (including SCI-EXPANDED 1970-present, SSCI 1970-present, AHCI 1975-present, CPCI-S 1990-present, CPCI-SSH 1990-present and ESCI 2015-present)

(incurable or advanced or inoperable or unresectable or metasta* or palliat* or terminal* or "stage 4" or "stage iv" or secondary or recurren*) Near/2 (cancer* or neoplas* or tumor* or tumour* or malignan* or carcinoma* or oncolog* or leukemi* or leukaemi* or lymphoma* or myeloma* or sarcoma*) (Topic) and wearable* or fitness tracker* or internet or web-based or digital health or digital intervention* or online intervention or mobile app or mobile apps or telemedicine or ambient assisted living or ambient intelligence or (smart Near/2 (home* or device* or applianc* or plug* or speaker*)) (Topic) and exercise* or sport* or diet* or lifestyle* or nutrition* or physical activity or sedentary (Topic)

## CENTRAL via The Cochrane Library

ID Search

#1 MeSH descriptor: [Neoplasms] explode all trees

#2 (cancer* or neoplas* or tumor* or tumour* or malignan* or carcinoma* or oncolog* or leukemi* or leukaemi* or lymphoma* or myeloma* or sarcoma*):ti,ab,kw

#3 #1 or #2

#4 (incurable or advanced or inoperable or unresectable or metasta* or palliat* or terminal* or "stage 4" or "stage iv" or secondary or recurren*):ti,ab,kw

#5 MeSH descriptor: [Palliative Care] explode all trees

#6 (((late or advanced or last or end or final) NEAR/3 (phase* or stage*))):ti,ab,kw

#7 #4 or #5 or #6

#8 #3 and #7

#9 MeSH descriptor: [Cancer Survivors] explode all trees

#10 (cancer* NEXT survivor*):ti,ab,kw

#11 #8 or #9 or #10

#12 MeSH descriptor: [Fitness Trackers] explode all trees

#13 MeSH descriptor: [Wearable Electronic Devices] this term only

#14 MeSH descriptor: [Smart Glasses] explode all trees

#15 MeSH descriptor: [Internet of Things] explode all trees

#16 (IoT or "Internet of things"):ti,ab,kw

#17 MeSH descriptor: [Accelerometry] explode all trees

#18 MeSH descriptor: [Artificial Intelligence] explode all trees

#19 MeSH descriptor: [Telemetry] 3 tree(s) exploded

#20 (((smart or connected) NEAR/3 (home* or device* or applianc* or plug* or sensor* or monitor*))):ti,ab,kw

#21 (acceleromet*):ti,ab,kw

#22 (((sensor* NEAR/3 (motion or door or smart or monitor*)) or (electronic NEAR/2 symptom* NEAR/2 monitor*))):ti,ab,kw

#23 (((frailty NEAR/2 monitor*) or ((computer* or electronic or technol*) NEAR/2 (assessment* or monitoring)))):ti,ab,kw

#24 (((non-instrusive or nonintrusive or passive or unobtrusive or remote*) NEAR/2 (sensor* or monitor*))):ti,ab,kw

#25 (ambient assisted living OR ambient intelligence):ti,ab,kw

#26 (real-time monitoring):ti,ab,kw

#27 (wearable*):ti,ab,kw

#28 ((sensor* NEAR/3 (network or wearable))):ti,ab,kw

#29 (inertial sensor*):ti,ab,kw

#30 ((((fitness or step* or activity) NEAR/2 (band* or tracker* or counter* or monitor*)) or fitbit*)):ti,ab,kw

#31 ((smart NEAR/2 (speaker* or watch or cloth* or bed* or ring*))):ti,ab,kw

#32 MeSH descriptor: [Internet] explode all trees

#33 ((online or internet) NEAR/2 intervention*):ti,ab,kw

#34 MeSH descriptor: [Telemedicine] 3 tree(s) exploded

#35 ((telehealth or telerehabilitation or telenursing or distance-based or broad-reach or (remote* NEAR/2 deliver*))):ti,ab,kw

#36 MeSH descriptor: [Mobile Applications] this term only

#37 MeSH descriptor: [Electronic Mail] 2 tree(s) exploded

#38 MeSH descriptor: [Videoconferencing] 1 tree(s) exploded

#39 MeSH descriptor: [Wireless Technology] 1 tree(s) exploded

#40 MeSH descriptor: [Cell Phone] 1 tree(s) exploded

#41 (telemedicine or mobile app* or smartphone* or mhealth or m-health or ehealth or e-health or sms or texting or text messag* or website* or web-based or web based or wireless or wearable* or (digital NEAR/2 (health or intervention*))):ti,ab,kw

#42 (tablet* or handheld*) NEXT computer*

#43 MeSH descriptor: [Video Games] explode all trees

#44 MeSH descriptor: [User-Computer Interface] explode all trees

#45 active NEAR/2 gam*

#46 (exergam* or nintendo wii or xbox kinect or wii fit):ti,ab,kw

#47 MeSH descriptor: [Computers, Handheld] explode all trees

#48 (((digital or voice) NEXT assistant*)):ti,ab,kw

#49 (google assistant or SIRI or Alexa):ti,ab,kw

#50 MeSH descriptor: [Speech Recognition Software] explode all trees

#51 (speech recognition software):ti,ab,kw

#52 MeSH descriptor: [Patient Portals] explode all trees

#53 {OR #12-#52}

#54 MeSH descriptor: [Exercise] explode all trees

#55 MeSH descriptor: [Exercise Therapy] explode all trees

#56 (physical activity or fitness or conditioning or exercis* or exerciz*):ti,ab,kw

#57 MeSH descriptor: [Sedentary Behavior] explode all trees

#58 (sedentary):ti,ab,kw

#59 MeSH descriptor: [Yoga] explode all trees

#60 MeSH descriptor: [Tai Ji] explode all trees

#61 ((tai ji or t'ai chi or tai chi or taiji or taijiquan)):ti,ab,kw

#62 (yoga or yogic):ti,ab,kw

#63 MeSH descriptor: [Qigong] explode all trees

#64 (qigong or qi gong or ch'i kung):ti,ab,kw

#65 ((holistic or mindful*) NEAR/2 movement):ti,ab,kw

#66 (active lifestyle*):ti,ab,kw

#67 MeSH descriptor: [Sports] explode all trees

#68 MeSH descriptor: [Diet] explode all trees

#69 MeSH descriptor: [Nutrition Therapy] explode all trees

#70 MeSH descriptor: [Diet Therapy] explode all trees

#71 ((diet* or nutrition* or lifestyle*)):ti,ab,kw

#72 MeSH descriptor: [Diet, Healthy] explode all trees

#73 {OR #54-#72}

#74 #11 and #53 and #73
