# Supplementary material for: Using digital technology to support wellbeing and independence among people living with incurable cancers: a systematic review
Source: Support Care Cancer. 2025 Jul 18;33(8):699. doi: 10.1007/s00520-025-09759-1 (PMC12274145; doi:10.1007/s00520-025-09759-1)
Supplement: Supplementary file 3 — Supplemental Three (DOCX 57.4 KB) [file 520_2025_9759_MOESM3_ESM.docx]

Table 1: illustrating the breakdown of quality appraisal scores and inter-rater reliability values for **quantitative** method studies.

| Author | Checklist Item | | | | | | | |
| --- | --- | --- | --- | --- | --- | --- | --- | --- |
|  | 1) Question or  objective sufficiently  described? | 2) Evident and  appropriate design | 3) Subject  selection | 4) Subject  characteristics | 5) Random  allocation | 6) Blinding of  investigators | 7) Blinding  of subjects | 8) Defined and  robust OMs |
| Asensio-Cuesta et al 2024 | 2 | 1 | 2 | 2 | N/A | N/A | N/A | 2 |
| Bade et al 2018 | 1 | 1 | 2 | 2 | 0 | 0 | 0 | 2 |
| Bade et al 2021 | 2 | 2 | 2 | 2 | 2 | 0 | 0 | 2 |
| Bergerot et al 2025 | 2 | 2 | 1 | 2 | N/A | N/A | N/A | 2 |
| Cheong et al 2018 | 2 | 1 | 2 | 0 | 0 | 0 | 0 | 2 |
| Cheville et al 2012 | 2 | 1 | 2 | 2 | 2 | 0 | 0 | 2 |
| Cheville et al 2019 | 2 | 2 | 2 | 2 | 2 | 2 | 0 | 2 |
| Coats et al 2020 | 2 | 2 | 2 | 2 | N/A | N/A | N/A | 2 |
| Crosby et al 2023 | 2 | 2 | 2 | 2 | N/A | N/A | N/A | 2 |
| Delrieu et al 2020 | 2 | 2 | 2 | 2 | N/A | N/A | N/A | 2 |
| Dorion et al 2017 | 2 | 2 | 1 | 2 | N/A | N/A | N/A | 2 |
| Evans et al 2021 | 2 | 2 | 2 | 2 | 2 | 0 | 0 | 2 |
| Hacker et al 2020 | 2 | 2 | 2 | 2 | 2 | 0 | 0 | 2 |
| Kenfield et al 2021 | 1 | 2 | 2 | 2 | 2 | 2 | 0 | 2 |
| Keum et al 2021 | 2 | 2 | 2 | 2 | 2 | 0 | 0 | 2 |
| Kim et al 2018 | 2 | 2 | 2 | 2 | 1 | 0 | 0 | 2 |
| Lee et al 2024 | 2 | 2 | 2 | 2 | 1 | 0 | 0 | 2 |
| Longacre et al 2020 | 2 | 2 | 2 | 2 | N/A | N/A | N/A | 2 |
| Low et al 2023 | 2 | 2 | 2 | 2 | 2 | 0 | 0 | 2 |
| Park et al 2019 | 2 | 1 | 2 | 2 | N/A | N/A | N/A | 2 |
| Phillps et al 2024 | 2 | 2 | 2 | 2 | 2 | 2 | 2 | 2 |
| Purdy et al 2022 | 2 | 2 | 2 | 2 | N/A | N/A | N/A | 2 |
| Schmitz et al 2021 | 2 | 2 | 2 | 1 | 2 | 0 | 0 | 2 |
| Schmitz et al 2023 | 2 | 2 | 2 | 2 | 1 | 1 | 0 | 2 |
| Shachar et al 2023 | 2 | 2 | 2 | 2 | N/A | N/A | N/A | 2 |
| Soh et al 2018 | 1 | 2 | 2 | 2 | N/A | N/A | N/A | 2 |
| Wallace et al 2025 | 2 | 2 | 2 | 2 | 2 | 1 | 2 | 2 |
| Wang et al 2021 | 2 | 2 | 2 | 2 | N/A | N/A | N/A | 2 |
| Wolff et al 2024 | 2 | 2 | 2 | 2 | 1 | 2 | 2 | 2 |

Table 1 (cont.): illustrating the breakdown of quality appraisal scores and inter-rater reliability values for **quantitative** method studies.

| Author | Checklist Item | | | | | | Score (%) | Inter-Rater Reliability (%) |
| --- | --- | --- | --- | --- | --- | --- | --- | --- |
|  | 9) Sample size | 10) Analysis described  and appropriate | 11) Estimate  of variance | 12) Controlled  for confounding | 13) Sufficient  Results | 14) Results match  Conclusions? |  |  |
| Asensio-Cuesta et. al. (2024) | 1 | 2 | 2 | 2 | 2 | 2 | 86 | 100 |
| Bade et. Al. (2018) | 0 | 2 | 2 | 0 | 2 | 2 | 57 | 100 |
| Bade et al 2021 | 2 | 2 | 2 | 2 | 2 | 2 | 86 | 100 |
| Bergerot et al 2025 | 2 | 2 | 1 | 2 | 2 | 2 | 91 | 100 |
| Cheong et al 2018 | 0 | 2 | 2 | 0 | 2 | 2 | 43 | 100 |
| Cheville et al 2012 | 2 | 2 | 2 | 2 | 2 | 2 | 79 | 93 |
| Cheville et al 2019 | 1 | 2 | 2 | 2 | 2 | 2 | 89 | 93 |
| Coats et al 2020 | N/A | 2 | 2 | 2 | 2 | 2 | 100 | 93 |
| Crosby et al 2023 | 2 | 2 | 2 | 2 | 2 | 2 | 89 | 100 |
| Delrieu et al 2020 | 2 | 2 | 2 | 2 | 2 | 2 | 100 | 100 |
| Dorion et al 2017 | 2 | 2 | 2 | 1 | 2 | 2 | 91 | 93 |
| Evans et al 2021 | 2 | 2 | 2 | 2 | 2 | 2 | 86 | 100 |
| Hacker et al 2020 | 2 | 2 | 2 | 2 | 2 | 2 | 86 | 100 |
| Kenfield et al 2021 | 1 | 2 | 2 | 2 | 2 | 2 | 92 | 100 |
| Keum et al 2021 | 2 | 2 | 2 | 2 | 2 | 2 | 86 | 100 |
| Kim et al 2018 | 2 | 2 | 2 | 2 | 2 | 2 | 93 | 100 |
| Lee 2024 | 1 | 2 | 2 | 2 | 2 | 2 | 86 | 92 |
| Longacre et al 2020 | 1 | 2 | 2 | 2 | 2 | 2 | 95 | 100 |
| Low et al 2023 | 1 | 2 | 2 | 2 | 2 | 2 | 82 | 100 |
| Park et al 2019 | 2 | 2 | 2 | 2 | 2 | 2 | 95 | 100 |
| Phillps et al 2024 | 2 | 2 | 2 | 2 | 2 | 2 | 100 | 100 |
| Purdy et al 2022 | 2 | 2 | 2 | 2 | 2 | 2 | 89 | 100 |
| Schmitz et al 2021 | 1 | 2 | 2 | 2 | 2 | 2 | 79 | 100 |
| Schmitz et al 2023 | 1 | 2 | 2 | 2 | 2 | 2 | 82 | 100 |
| Shachar et al 2023 | 1 | 2 | 2 | 2 | 2 | 1 | 91 | 100 |
| Soh et al 2018 | N/A | 2 | 2 | N/A | 2 | 2 | 94 | 100 |
| Wallace et al 2025 | 2 | 2 | 2 | 1 | 2 | 2 | 93 | 79 |
| Wang et al 2021 | 2 | 2 | 2 | 2 | 2 | 2 | 79 | 100 |
| Wolff et al 2024 | 0 | 2 | 2 | 2 | 2 | 2 | 89 | 86 |
