# Supplementary material for: Using digital technology to support wellbeing and independence among people living with incurable cancers: a systematic review
Source: Support Care Cancer. 2025 Jul 18;33(8):699. doi: 10.1007/s00520-025-09759-1 (PMC12274145; doi:10.1007/s00520-025-09759-1)
Supplement: Supplementary file 4 — Supplement Four (DOCX 72.2 KB) [file 520_2025_9759_MOESM4_ESM.docx]

Supplement Five: Detailed Efficacy Outcomes

| **Source** | **Quality of Life** | **Physical Activity/function** | **Fatigue and/or Pain** | **Anxiety and/or Depression** | **Dyspnoea, Sleep, and/or Nutrition** |
| --- | --- | --- | --- | --- | --- |
| Asensio-Cuesta et. al. (2024) | EORTC QLQ-C30 – isolated results not given but comments state correlations with sensor data and that QoL and functional status varied after treatment. | ECOG and Mobile sensor – Patient 1 showed a deterioration in ECOG directly after first treatment returning to baseline after the second cycle, Patient 2 showed improved ECOG. | Frequently reported symptoms for both patients  were tiredness and pain. Participant 1’s pain disappeared after one cycle of chemotherapy. | NM | Participant 1’s dyspnoea disappeared after one cycle of chemotherapy. |
| Bade et. Al. (2018) | NM | Daily step count:  Weekly phone call group (mean):  Baseline: 5128  Week 3: 5247, ES=0.02  Twice Daily text group:  Baseline: 4906  Week 3: 5241, ES 0.05 | NM | NM | NM |
| Bade et al 2021 | EORTC QLQ-C30: QoL Summary Score M(SD)  Week 1 to 12: IG: 2.28 (2.18), UC: 0.97 (2.22)  Between group difference M(SD): 1.31 (3.02), p=0.668 | PA min/week at 3 months M(SD)  IG: +123 (212)  UC: +35 (SD 103)  p = 0.051  EORTC Physical Functioning change, M(SD):  IG: 4.11 (2.50),  UC: 4.74 (2.54)  Between group difference: -0.64 (3.41), p=0.853  Change in EORTC Role Functioning M(SD)  IG: 8.09 (5.33)  UC: -8.95 (5.45)  p=0.0222 | EORTC Pain score change, M(SD):  IG: 1.67 (4.77)  UC 1.26 (4.87)  Between group difference: 0.41 (6.28), p=0.948.  EORTC Fatigue score change M(SD):  IG: -4.43 (4.85)  UC: 0.75 (4.97)  Between group difference: -5.18 (6.88), p=0.456 | Change in depression (PHQ-9) score M(SD):  IG: -0.83 (0.65)  UC: 0.31 (0.68)  Between group difference: -1.14 (0.88), p=0.203 | Change in MMRC Dyspnea Scale score M(SD):  IG: -0.04 (0.15)  UC -0.07 (0.15)  Between group difference: 0.03 (0.20), p=0.889  Change in EORTC Dsypena score M(SD):  IG: -6.19 (4.88)  UC: -13.31 (6.62)  Between group difference: 13.31 (6.62) p=0.051  Change in EORTC Appetite Loss Scale score M(SD):  IG: -4.16 (6.60)  UC: 4.95 (6.73)  Between group difference: -9.11 (8.50), p=0.290  Change in EORTC Insomnia score M(SD):  IG: 0.58 (6.37)  UC: -3.49 (6.52)  Between group difference: 4.07 (8.71), p=0.643. |
| Bergerot et al 2025 | FACT-G Baseline Mean 83.2; Post Mean 94.4, p=0.001 | NM | Edmonton Symptom Assessment System (ESAS)  Pain – Baseline Mean: 1.6, Post Mean: 0.7, P = 0.001  Fatigue – Baseline Mean: 3.8, Post Mean: 1.3, P = 0.001 | Edmonton Symptom Assessment System (ESAS)  Depression – Baseline Mean: 2.6, Post Mean: 0.8, P = 0.001  Anxiety – Baseline Mean: 3.2, Post Mean: 1.2, P = 0.001 | Edmonton Symptom Assessment System (ESAS)  Drowsiness – Baseline Mean: 1.8, Post Mean: 0.4, P = 0.001  Appetite – Baseline Mean: 2.2, Post Mean: 0.6, P = 0.001  Nausea – Baseline Mean: 0.8, Post Mean: 0.2, P = 0.01  Shortness of Breath – Baseline Mean: [not provided], Post Mean: [not provided], P = 0.78 |
| Cheong et al 2018 | EORTC QLQ-C30 Global Health Status M(SD):  Baseline: 58.11 (22.75)  6 weeks: 60.77 (22.83)  12 weeks: 57.11 (21.29)  p=0.271 | Handgrip strength (kg, M(SD)):  Baseline: 32.02 (10.91)  6 weeks: 32.69 (10.54)  12 weeks: 32.61 (11.65)  p=0.287  30-second Chair Stand Test (number of sit-to-stands, M(SD)):  Baseline: 17.30 (6.21)  6 weeks: 19.06 (6.56)12 weeks: 20.92 (7.12)  p<.001  2-minute Walk Test (meters, M(SD)):  Baseline: 166.76 (29.60)  6 weeks: 174.41 (31.47)12 weeks: 182.19 (33.09)  p<0.001  Total METs (IPAQ, MSD)):  Baseline: 2450.85 (2773.20)  6 weeks: 3858.88 (5700.29)  12 weeks: 3036.54 (3667.38)  p=0.118  EORTC QLQ-C30, Physical Functioning M(SD):  Baseline: 77.20 (17.68)  6 weeks: 78.42 (17.03)  12 weeks: 77.95 (18.79)  p=0.695 | EORTC QLQ-C30 Pain M(SD):  Baseline: 18.66 (24.19)  6 weeks: 16.00 (21.12)  12 weeks: 15.55 (23.93)  p=0.471  EORTC QLQ-C30 Fatigue M(SD): Baseline: 31.62 (23.19)  6 weeks: 30.81 (22.26)  12 weeks: 26.07 (21.80)  p=0.007 | NM | EORTC QLQ-C30 Dyspnea M(SD):  Baseline: 18.22 (22.11)  6 weeks: 20.00 (25.11)  12 weeks: 18.66 (20.67)  p=0.838  EORTC QLQ-C30 Insomnia M(SD):  Baseline: 32.00 (31.21)  6 weeks: 30.66 (25.56)  12 weeks: 26.66 (25.70)  p=0.321  EORTC QLQ-C30 Appetite Loss M(SD):  Baseline: 27.11 (28.83)  6 weeks: 20.88 (27.27)  12 weeks: 20.00 (25.70)  p=0.085 |
| Cheville et al 2012 | FACT-G M (95% CI):  IG: 1.07 (-5.97 to 8.83)  CG: 0.12 (-3.19 to 3.74) p=0.54 | Activity Short Form M(95%CI):  IG: 1.56 (-0.72, 3.84)  CG: 0.94 (-1.26, -3.14)  p=0.74  Mobility Short Form M(95%CI):  IG: 4.88 (2.96, 6.80)  CG: 0.23 (-1.76, 2.22)  p=0.002 | FACT-F M(95%CI):  IG: 4.46 (0.81, 8.11)  CG: -0.79 (-4.26, 2.67)  p=0.03  Pain  IG: Mean difference = -0.62 (95% CI: -1.66 to 0.43  CG: Mean difference = -0.50 (95% CI: -1.25, 0.25), P =0.87) | NM | Sleep Quality M(95%CI):  IG: 1.46 (0.70, 2.22)  CG: -0.10 (-0.74, 0.54)  p=0.002 |
| Cheville et al 2019 | EQ5D – 3L  Telerehabilitation Arm 2: Significant improvement (effect size = 0.23, coefficient = 0.04 [95% CI: 0.004 to 0.07], p = 0.01).  Telerehabilitation + Pain Management Arm 3: No significant improvement (effect size = 0.16, coefficient = 0.16 [95% CI: −0.01 to 0.06], p = 0.08). | AM-PAC-CAT Basic  Arm 2 (Telerehabilitation): Effect Size = 0.23. Coefficient (95% CI) = 1.30 (0.08 to 2.35). p-value = 0.03  Arm 3 (Telerehabilitation + Pain Management): Effect Size = 0.09. Coefficient (95% CI) = 0.48 (−0.67 to 1.63). p-value = 0.41 | Pain interference M(95%CI):  Arm 2: −0.4 (−0.78, −0.09), p=.01  Arm 3: −0.4 (−0.79, −0.10), p=0.01  Average Pain Intensity M(95%CI):  Arm 2: −0.4 (−0.78, −0.07), p=0.02  Arm 3: −0.5 (−0.84, −0.11), p=0.006 | NM | NM |
| Coats et al 2020 | EORTC QLQ-30  No significant changes | TUG (Mean ± SD): Δ 0.38 ± 0.47 s, p = 0.49  6MWT (Mean ± SD): Δ 40 ± 20 m, p = 0.01  TST (Mean ± SD): Δ 3.0 ± 0.2 s, p = 0.05  Quadriceps muscle peak torque (Mean ± SD): Δ 1.2 ± 18.7 Nm, p = 0.97  Quadriceps muscle total work (Mean ± SD): Δ 40.4 ± 374.0 Nm, p = 0.94  Quadriceps muscle fatigue index (Mean ± SD): Δ 8.21 ± 0.14%, p = 0.98  Weight (Mean ± SD): Δ 0.86 ± 3.5 Kg, p = 0.92  Lean body mass (Mean ± SD): Δ 0.22 ± 4.06 Kg, p = 0.97  Fat body mass (Mean ± SD): Δ 0.64 ± 1.0 Kg, p = 0.8  VO₂peak during CPET (Mean ± SD): Δ 0.68 ± 1.96 mL·kg⁻¹·min⁻¹, p = 0.74 | NM | French Canadian version of the Hospital Anxiety and Depression Scale (HADS) – data not reported | NM |
| Crosby et al 2023 | EORTC-QLQ-30 Global Health Status (Median (IQR)):  Baseline: 66.7 (47.9–83.3)  Post-intervention: 75.0 (58.3–83.3)  p=0.888 | EORTC-QLQ-30 Physical Functioning (Median (IQR)):  Baseline: 93.3 (65.0–100.0)  Post-intervention: 96.7 (70.0–100.0)  p=0.315  ABC Scale (Median (IQR)):  Baseline: 140.5 (115.0–150.0)  Post-intervention: 147.0 (122.3–149.0)  p=0.192  IPAQ-SF Physical Activity Levels (MET-min/week; (Median (IQR)):  Baseline: 516 (363–1292) Post-intervention: 1374 (435–1763)  p=0.047  IPAQ-SF Sedentary Behaviour (min/week; (Median (IQR)):  Baseline: 3225 (2363–4500)  Post-intervention: 2550 (1650–3450)  p=0.107  Two-minute step test (steps; M(SD)):  Baseline: 88.3 (27.9)  Post-intervention: 103.8(29.5)  Between group difference M(95%CI): 15.5 (10.5–20.5) p<0.001    30-second push-up test (reps, M(SD)):  Baseline: 10.1 (± 4.8)  Post-intervention: 14.1 (± 3.6)  Between group difference M(95%CI): 4.0 (1.2–6.8)  p=0.010  Chair rise test (sec, M(SD)):  Baseline: 12.5 (± 3.3)  Post-intervention: 9.6 (± 2.8) Between group difference: −2.9 (−4.7, −1.1)  p=0.006  Static balance (sec; median (IQR)):  Baseline: 190.2 (129.3–201.3)  Post-intervention: 197.8 (160.6–219.2)  p=0.007 | EORTC-QLQ-30 Fatigue, (Median (IQR)):  Baseline: 44.4 (22.2–66.7)  Post-intervention: 33.3 (19.4–47.2)  p=0.440  EORTC-QLQ-30 Pain (Median (IQR)):  Baseline: 25.0 (12.5–25.0)  Post-intervention: 16.7 (0.0–37.5)  p=0.786 | NM | EORTC-QLQ-30 Dyspnoea, (Median (IQR)):  Baseline: 0.0 (0.0–33.3)  Post-intervention: 16.7 (0.0–41.7), p=0.084  EORTC-QLQ-30 Insomnia, (Median (IQR)):  Baseline: 33.3 (25.0–66.7)  Post-intervention: 33.3 (0.0–66.7), p=1.000  EORTC-QLQ-30 Appetite Loss, (Median (IQR)):  Baseline: 0.0 (0.0–33.3)  Post-intervention: 0.0 (0.0–8.3), p=0.157 |
| Delrieu et al 2020 | EORTC-QLQ-30 Global Health Score M(SD):  Baseline: 62.7 (20.6)  End of Study: 63.5 (23.2) p=0.74 | EORTC-QLQ-30 Physical Functioning, M(SD):  Baseline: 76.3 (22.4),  End of Study: 82.0 (17.1) p=0.17  6-minute walking test (6MWD; M(SD)):  Baseline: 451.6 (99.7)  End of Study: 482.6 (106.3) p<0.001  VO2peak (mL/kg/min; M(SD)):  Baseline: 13.7 (4.4)  End of Study: 13.5 (6.0)  p=0.71  Handgrip Strength, Left (kg, M(SD)):  Baseline: 30.1 (35.3)  End of Study: 24.1 (4.4)  p=0.25  Handgrip Strength, Right (kg, M(SD)):  Baseline: 26.2 (6.1)  End of Study: 26.2 (4.3)  p=0.17  Isometric Quad strength (N):  Baseline: 194.2 (69.1)  End of Study: 236.4 (78.6)  p<0.001 | EORTC QLQ-C30 Fatigue:  Baseline: 44.2 (± 27.4), End of Study: 36.9 (± 27.6), P-value: 0.08  Fatigue (Piper Scale; n (%)):  Yes:  Baseline: 30 (61%)  End of Study: 28 (61%)  p>0.99  No:  Baseline: 19 (39%)  End of Study: 18 (39%)  p>0.99  EORTC QLQ-C30 Pain, M(SD):  Baseline: 35.1 (31.6)  End of Study: 25.4 (26.3)  p=0.29 | NM | EORTC QLQ-C30 Dyspnea:  Baseline: 28.5 (± 30.7), End of Study: 22.7 (± 26.7), P-value: 0.70  EORTC QLQ-C30 Insomnia, M(SD):  Baseline: 40.3 (35.0)  End of Study: 28.8 (29.3)  p=0.37  EORTC QLQ-C30 Appetite Loss, M(SD):  Baseline: 20.8 (27.2)  End of Study: 9.9 (21.1)  p=0.02 |
| Dorion et al 2017 | EORTC QLQ-C30  Questionnaire was completed by 6 patients on day 7. There was no observable correlation between an improvement in the QOL and the number of steps | Step count via Activity Tracker.  Compared to pre-RT, the patients after palliative RT took 30% less daily steps, which was significant. A reduced activity level (≤ 7800 vs. > 7800 average steps per day) prior to RT was significantly associated with overall survival.  Performance Status – KPS.  The general performance of the patients at baseline ranged from 70-90 KPS. KPS prior to palliative RT was not prognostic. | Pain – SF-BPI  Pain flare was observed in 5 patients.  8 out of the 9 who responded to the SF-BPI had a clinically important reduction in pain by day 7. There was no significant correlation between reduction in pain level and change in daily activity level | NM | NM |
| Evans et al 2021 | EORTC-QLQ-30 Global Health Status, M(SD):  Baseline:  EG: 62.7 (22.3)  CON: 71.9 (15.0)  Follow-up:  EG: 68.4 (22.0)  CON: 64.5 (22.2)  Adjusted between group difference M(95%CI):  9.3 (-3.7–22.4), p=0.24 | MVPA (min/day):  Baseline:  EG: 30.57 (22.0)  CON: 38.4 (22.2)  Follow-up:  EG: 35.1 (23.6)  CON: 32.0 (22.7  Adjusted between group difference M(95%CI): 10.0 (1.3–18.6), p=0.01  EORTC-QLQ-30 Physical Functioning, M(SD):  Baseline:  EG: 84.1 (16.2)  CON: 90.9 (12.0)  Follow-up:  EG: 85.9 (17.4)  CON: 87.0 (12.5)  Adjusted between group difference M(95%CI): 4.1 (-2.8–10.9), p=0.44 | EORTC-QLQ-30 Fatigue, M(SD):  Baseline:  EG: 36.3 (20.2)  CON: 31.6 (21.7)  Follow-up:  EG: 39.8 (19.4)  CON: 38.0 (23.0)  Adjusted between group difference M(95%CI): 2.5 (-7.2–12.2), p=0.56  EORTC-QLQ-30 Pain M(SD):  Baseline:  EG: 21.1 (24.7)  CON: 14.9 (19.2)  Follow-up:  EG: 24.6 (25.1)  CON: 21.9 (26.7)  Adjusted between group difference M(95%CI): -0.5 (-15.5–14.5), p=0.81 | Depression (HADS-D; M(SD)):  Baseline:  EG: 3.3 (3.1)  CON: 2.9 (1.9)  Follow-up:  EG: 3.1 (2.8)  CON: 4.1 (2.3)  Adjusted between group difference M(95%CI): -1.3 (-2.4–2.4), p=0.06  Anxiety (HADS-A; M(SD)):  Baseline:  EG: 2.9 (3.2)  CON: 4.4 (2.7)  Follow-up:  EG: 3.2 (3.4)  CON: 4.7 (3.3)  Adjusted between group difference M(95%CI): -0.2 (-1.7–1.2), p=0.74 | Dyspnoea M(SD):  Baseline:  EG: 14.0 (16.9)  CON: 15.8 (17.1)  Follow-up:  EG: 19.3 (16.9)  CON: 19.3 ± 16.9)  Adjusted between group difference: -2.6 (-14.4–9.2), p=0.40  Appetite Loss, M(SD):  Baseline:  EG: 10.5 (15.9)  CON: 1.8 (7.6)  Follow-up:  EG: 13.2 (27.7)  CON: 5.3 (16.7)  Adjusted between group difference M(95%CI): -11.7 (-27.5–5.1), p=0.18  Insomnia, M(SD):  Baseline:  EG: 36.8 (27.0)  CON: 24.6 (24.4)  Follow-up:  EG: 29.8 (29.7)  CON: 29.8 (29.2)  Adjusted between group difference M(95%CI): -1.3 (-13.9–26.2), p=0.27  Sleep Index (PSQI; M(SD)):  Baseline:  EG: 7.2 (2.9)  CON: 6.9 (3.3)  Follow-up:  EG: 11.5 (3.7)  CON: 10.7 (3.1)  Adjusted between group difference M(95%CI): 0.6 (-1.4–2.6), p=0.10 |
| Hacker et al 2020 | Global Health Status QoL, M(SD):  Overall Group (QoL Score): Baseline: 64.0 (17.9)  Percentage Change: 2.2%  Post-intervention: 65.4 (17.3)  STEPS Group:  Baseline: 64.7 (16.8)  Post-intervention: 68.6 (18.2) Percent Change: 6.0%  UC Group:  Baseline: 63.3 (19.6)  Post-intervention: 61.7 (16.0)  Percent Change: -2.5 | Physical Functioning, M(SD):  Overall Group:  Baseline: 81.0 (14.7)  Post-intervention: 76.5 (20.5)  Percent Change: -5.6%,  STEPS Group:  Baseline: 82.0 (15.9)  Post-intervention: 76.4 (19.2)  Percent Change: -6.8%,  UC Group:  Baseline: 80.0 (13.6)  Post-intervention: 76.4 (22.5)  Percent Change: -4.5%  Hand Grip Strength (Right, kg, M(SD)):  Overall Group:  Baseline: 32.0 (11.5)  Post-intervention: 29.6 (11.5)  Percent Change: -7.5%.  STEPS Group:  Baseline: 30.0 (11.6)  Post-intervention: 27.8 (11.5)  Percent Change: -7.3%  UC Group:  Baseline: 34.1 (11.4)  Post-intervention: 31.5 (11.7)  Percent Change: -7.6%  Hand Grip Strength (Left, kg, M(SD)):  Overall Group:  Baseline: 31.0 (10.3)  Post-intervention: 27.7 (10.6)  Percent Change: -10.6%  STEPS Group:  Baseline: 30.5 (10.6)  Post-intervention: 26.0 (10.0)  Percent Change: -14.8%  UC Group:  Baseline: 31.6 (10.3)  Post-intervention: 29.6 (11.2)  Percent Change: -6.3%  Arm Curl Test (Reps in 30s, M(SD)):  Overall Group:  Baseline: 17.8 (5.1)  Post-intervention: 17.9 (8.4)  Percent Change: 0.6%  STEPS Group:  Baseline: 17.8 (5.1)  Post-intervention: 17.9 (8.4)  Percent Change: 0.6%  UC Group:  Baseline: 18.3 (4.8)  Post-intervention: 17.9 (9.2)  Percent Change: -2.2%  Timed Stair Climb (s, M(SD)):  Overall Group:  Baseline: 6.5 (2.6)  Post-intervention: 7.6 (3.3)  Percent Change: 16.9%  STEPS Group:  Baseline: 6.3 (2.8)  Post-intervention: 8.0 (3.8)  Percent Change: 27.0%  UC Group:  Baseline: 6.6 (2.4)  Post-intervention: 7.3 (2.9)  Percent Change: 10.6%  Timed Up and Go Test (s, M(SD)):  Overall Group:  Baseline: 8.3 (3.1)  Post-intervention: 9.1 (3.8)  Percent Change: 9.6%  STEPS Group:  Baseline: 8.2 (3.7)  Post-intervention: 9.1 (3.8)  Percent Change: 9.6%  UC Group:  Baseline: 8.3 (2.6)  Post-intervention: 9.0 (3.7)  Percent Change: 8.4%  30-s Chair Stand Test (Reps in 30s, M(SD)):  Overall Group:  Baseline: 10.9 (4.4)  Post-intervention: 10.9 (5.6)  Percent Change: -0.0%  STEPS Group:  Baseline: 11.0 (4.2)  Post-intervention: 9.9 (6.3)  Percent Change: -10.0%  UC Group:  Baseline: 10.7 (4.8)  Post-intervention: 11.9 (4.8)  Percent Change: 11.2% | Pain (EORTC; M(SD)): Overall Group: 31.8 (26.2)  Post-intervention: 19.8 (19.6)  Percent Change: -37.7  STEPS Group:  Baseline: 31.4 (29.4)  Post-intervention: 18.6 (19.4)  Percent Change: -40.8%  UC Group:  Baseline: 32.2 (23.1)  Post-intervention: 21.1 (20.4)  Percent Change: -34.5%  Overall Fatigue (Chalder Fatigue Scale, M(SD)):  Baseline: 27.0 (4.2)  Post-intervention: 25.5 (5.9)  Percentage Change: - 5.6  STEPS Group:  Baseline: 26.6 (4.8)  Post-intervention: 26.1 (5.4)  Percent Change: -1.9%  UC Group:  Baseline: 27.3 (3.6)  Post-intervention: 24.9 (6.6)  Percent Change: -8.8%  Physical Fatigue (Chalder Fatigue Scale, M(SD)):  Overall group:  Baseline: 17.7 (4.0)  Post-intervention: 17.0 (4.6)  Percentage Change: -4.0  STEPS Group:  Baseline: 17.4 (4.8)  Post-intervention: 17.4 (4.5)  Percent Change: 0.0%  UC Group:  Baseline: 18.0 (3.0)  Post-intervention: 16.7 (5.0)  Percent Change: -7.2%  Mental Fatigue (Chalder Fatigue Scale, M(SD)):  Overall Group:  Baseline: 9.3 (1.4)  Post-intervention: 8.4 (2.0)  Percentage Change: -9.7%  STEPS Group:  Baseline: 9.3 (1.5)  Post-intervention: 8.7 (1.9) Percent Change: -6.5%  UC Group:  Baseline: 9.3 (1.3)  Post-intervention: 8.1 (2.3)  Percent Change: -12.9%  Fatigue (EORTC; M(SD)):  Overall Group:  Baseline: 36.5 (20.8)  Post-intervention: 35.4 (21.0)  Percent Change: -3%  STEPS Group:  Baseline: 33.3 (20.0)  Post-intervention: 36.6 (17.5)  Percent Change: 9.9%  UC Group:  Baseline: 72.2 (25.7)  Post-intervention: 40.0 (21.7)  Percent Change: -14.8%  Fatigue (PROMIS, M(SD)):  Overall Group:  Baseline: 53.6 (10.0)  Post-intervention: 51.2 (10.0)  Percentage Change: -4.5%  STEPS Group:  Baseline: 53.7 (11.5)  Post-intervention: 52.8 (9.6)  Percentage Change: -1.7%  UC Group:  Baseline: 53.5 (8.5)  Post-intervention: 49.4 (10.5)  Percentage Change: -7.7% | Anxiety (M(SD)):  Overall group:  Baseline: 50.4 (9.7)  Post-intervention: 44.9 (8.2)  Percentage Change: -10.9%STEPS Group:  Baseline: 50.1 (9.6)  Post-intervention: 46.5 (8.9)  Percentage Change: -7.2%  UC Group:  Baseline: 50.6 (10.1)  Post-intervention: 43.0 (7.2)  Percentage Change: -15.0%  Depression (M(SD)):  Overall Group:  Baseline: 46.0 (6.8)  Post-intervention: 43.8 (7.02)  Percentage Change: -4.8%STEPS Group:  Baseline: 46.6 (8.1)  Post-intervention: 45.3 (8.2)  Percentage Change: -2.8%  UC Group:  Baseline: 45.4 (5.1)  Post-intervention: 42.2 (5.4)  Percentage Change: -7.0% | Total Sleep Time (M(SD)):  Overall Group:  Baseline: 440.2 (81.6)  Post-intervention: 454.1 (100.6)  Percentage Change: 3.2%STEPS Group:  Baseline: 454.4 (72.1)  Post-intervention: 484.4 (113.6)  Percentage Change: 6.6%  UC Group:  Baseline: 403.7 (66.9)  Post-intervention: 418.2 (83.2)  Percentage Change: 3.6%  Dyspnoea (M(SD)):  Overall Group:  Baseline: 16.7 (25.4)  Post-intervention: 15.6 (25.4)  Percentage Change: -6.6%  STEPS Group:  Baseline: 17.6 (26.7)  Post-intervention: 15.7 (23.9)  Percentage Change: -10.8%  UC Group:  Baseline: 15.6 (24.8)  Post-intervention: 15.6 (27.8)  Percentage Change: -0.0%  Appetite Loss (M(SD)):  Overall Group:  Baseline: 12.5 (20.3)  Post-intervention: 20.8 (33.6)  Percentage Change: 66.4%  STEPS Group:  Baseline: 15.7 (20.8)  Post-intervention: 31.4 (39.9)  Percentage Change: -10%  UC Group:  Baseline: 8.9 (19.8)  Post-intervention: 8.9 (19.8)  Percentage Change: 0%  Sleep Disturbance (EORTC QLQ-C30, M(SD)):  Overall Group:  Baseline: 46.9 (29.2)  Post-intervention: 25.0 (28.1)  Percent Change: -46.7%  STEPS Group:  Baseline: 45.1 (33.2)  Post-intervention: 29.4 (33.1)  Percent Change: -34.8%  UC Group:  Baseline: 48.9 (24.8)  Post-intervention: 20.0 (21.1)  Percent Change: -59.1%  Sleep Disturbance (PROMIS, M(SD)):  Overall Group:  Baseline: 57.5 (8.4)  Post-intervention: 51.0 (9.6)  Percent Change: -11.3%  STEPS Group:  Baseline: 57.6 (8.5)  Post-intervention: 52.4 (11.4)  Percent Change: -9.0%  UC Group:  Baseline: 57.5 (8.4)  Post-intervention: 48.3 (7.1)  Percent Change: -16.0% |
| Kenfield et al 2021 | NM | Timed fitness measurements included: stair climb;  400-m walk; repeated sit-to-stand.  For resistance testing and initial training, 1-RM tests included the following exercises:  chest press; leg press or extension; seated row.  The resistance arm improved more in the 1-RM tests than the other two arms, while the aerobic arm had greater changes in the steep ramp test performed on the bike and the 400-m walk test than the other two arms. Resting heart rate was modestly reduced in the resistance and aerobic arms and modestly increased in the control arm. | Fatigue and bone pain – VAS at each exercise session.  Men in both arms reported no bone pain and low fatigue levels during exercise, with the aerobic arm reporting slightly more fatigue (median = 4) than the resistance arm (median = 3). | NM | NM |
| Keum et al 2021 | EORTC QLQ-C30 (30 items)  GHS (Global health Status) and QoL scale  There was no statistically significant difference in the EORTC QLQ score between groups. On the GHS and QoL scale, there was a statistically significant improvement Noom users compared to non-Noom users. | Performance status (ECOG).  There were no differences in ECOG PS pre and post treatment in the two groups.  Steps recorded (steps/week, M(SD)):  Noom users: 17,168.23 (20,718.02)  Above average users: 23,999.58 (24,595.55)  Below average users: 7,409.17 (6,951.79) Pre-post changes above average and below average users; p=0.02  Skeletal muscle index Decrease During Chemotherapy (cm²/m², M(SD):  Noom users:  Pre: 49.08 (12.27) Post: 46.08 (10.55),  Percent change: -3.27%  Non-Noom users:  Pre: 50.60 (9.05)  Post: 42.97 (8.12)  Percent change: -13.96%  p=0.11 | NM | NM | Nutritional Status (PG-SGA, M(SD)).  All study participants showed a significant improvement in the nutritional status according to the PG-SGA In per-protocol analysis, above average users showed a significant improvement in the PG-SGA score.  Total protein intake (g/kg/day, M(IQR)):  Noom users: 1 (0.6–1.4)  Above average users: 1.3 (0.9–1.6)  Below average users: 1 (0.5–1), p=0.02  Total energy intake (kcal/kg/day, M(IQR)):  Noom users: 19.9 (13.9–26.8)  Above average users: 25.2 (17.5–32.7)  Below average users: 17.7 (12.1–20.8). p=0.04  Weight loss (kg, M(SD)):  Noom users: -0.66 (4.31)  Above average users: 0.68 (4.80)  Below average users: -2.57 (2.76), p=0.10    Weight loss (%, M(SD)):  Noom users: -1.14 (7.55)  Above average users: 1.16 (8.31)  Below average users: -4.43 (5.21)  BMI change (kg/m², M(SD)):  Noom users: -0.21 (1.43)  Above average users: 0.21 (1.60)  Below average users: -0.81 (0.93), p=0.09 |
| Kim et al 2018 | Quality of Life M(SD):  Baseline:  Game Group: 77.5 (3.4)  Control Group: 76.8 (4.5)  p=0.48  3 weeks:  Game Group: 74.9 (3.5)  Control Group: 72.2 (5.3)  p=0.01  Differences:  Game Group: -2.6 (1.5)  Control Group: -4.6 (4.4)  p=0.01 | Physical side effects measured by questionnaires relating to commonly experienced side effects of anticancer medications.  The game group reported positive usefulness in overcoming chemotherapy side effects (73.9%).  The game group also showed improved compliance to medications and lower rates of physically adverse events, nausea, fatigue, and numbness in the hand or foot  . | NM | Beck’s Depression Index M(SD):  Baseline:  Game Group: 13.1 (3.5)  Control Group: 12.4 (5.6)  p=0.51  3 weeks:  Game Group: 15.7 (3.7)  Control Group: 14.9 (5.2)  p=0.50  Differences:  Game Group: 2.6 (1.1)  Control Group: 2.6 (1.7)  p=0.99  State Anxiety M(SD):  Baseline:  Game Group: 37.4 (3.8)  Control Group: 37.9 (3.3)  p=0.43  3 weeks:  Game Group: 40.6 (3.6)  Control Group: 42.0 (3.8)  p=0.11  Differences:  Game Group: 3.4 (0.9)  Control: 4.1 (3.4)  p=0.21. | NM |
| Lee et al 2024 | PROMIS-Global Health 10 Quality of Life  Physical Health  Baseline: 46.7 ± 7.3, Follow-up: 49.0 ± 5.5, p = 0.020  Mental Health  Baseline: 49.0 ± 4.3, Follow-up: 50.3 ± 5.5, p = 0.113 | Godin Leisure-Time Physical Activity Score  Baseline: 18.6 ± 18.7, Follow-up: 33.1 ± 24.3, p = 0.001  6-Minute Walk Test (m)  Baseline: 499.8 ± 98.4, Follow-up: 523.2 ± 107.3, p = 0.271  30-s Sit to Stand Test (repetitions)  Baseline: 12.2 ± 4.0, Follow-up: 14.0 ± 3.8, p = 0.046  Sit and Reach Test (cm)  Baseline: –1.2 ± 11.4, Follow-up: –0.2 ± 11.2, p = 0.359  Back Scratch Test (cm)  Baseline: –7.8 ± 16.9, Follow-up: –10.6 ± 13.1, p = 0.282  30-s Arm Curl Test, Right (repetitions)  Baseline: 15.6 ± 3.6, Follow-up: 16.7 ± 4.9, p = 0.030  30-s Arm Curl Test, Left (repetitions)  Baseline: 16.6 ± 4.1, Follow-up: 18.0 ± 4.7, p = 0.041  Grip Strength, Right (kg)  Baseline: 12.3 ± 9.2, Follow-up: 15.0 ± 10.0, p = 0.013  Grip Strength, Left (kg)  Baseline: 10.7 ± 9.0, Follow-up: 13.6 ± 10.1, p = 0.003  8-Foot Up and Go Test (seconds)  Baseline: 6.2 ± 1.7, Follow-up: 6.2 ± 2.0, p = 0.846 | NM | NM | Dietary Intake  Healthy Eating Index  Baseline: 65.2 ± 10.1, Follow-up: 68.0 ± 9.6, p = 0.043  Daily Calorie Intake (kcal)  Baseline: 1465.4 ± 581.4, Follow-up: 1243.9 ± 801.2, p = 0.033  Carbohydrates (% kcal)  Baseline: 45.9 ± 9.1, Follow-up: 47.0 ± 7.2, p = 0.494  Fat (% kcal)  Baseline: 39.2 ± 6.9, Follow-up: 36.3 ± 6.6, p = 0.061  Protein (% kcal)  Baseline: 16.4 ± 2.4, Follow-up: 17.6 ± 3.5, p = 0.029  Whole Grains (% total grains)  Baseline: 15.7 ± 11.7, Follow-up: 18.1 ± 15.6, p = 0.372  Vegetables (cups/day)  Baseline: 1.5 ± 1.0, Follow-up: 1.4 ± 1.5, p = 0.735  Fruit (cups/day)  Baseline: 1.4 ± 1.4, Follow-up: 1.3 ± 1.0, p = 0.372  Saturated Fat (% kcal)  Baseline: 12.3 ± 3.1, Follow-up: 10.7 ± 2.8, p = 0.014  Added Sugar (% kcal)  Baseline: 9.2 ± 5.8, Follow-up: 9.6 ± 7.9, p = 0.736 |
| Longacre et al 2020 | EQ-5D-3L QALYs  EQ-5D-3L not reported in write up, discussion states tele-rehabilitation improved quality of life and physical function.  The mean QALY gain in arm B was 0.01 (3.65 days of the equivalent of perfect health) and in arm C was 0.0075 (2.74 days) | NM | NM | NM | NM |
| Low et al 2023 | Quality of Life:  Time effect: Ftime=21.4, p<0.001.  Group effect: Fgroup=0.3, p=0.60. | Physical Symptoms: Time effect: Ftime = 24.0, P < 0.001. Group effect: Fgroup = 0.1, P = 0.76. Group × Time effect: Fgroup×time = 0.9, P = 0.41. Outcome: Worsening physical symptoms were reported after surgery. | NM | Depressive Symptoms: Time effect: Ftime = 10.9, P < 0.001. Group effect: Fgroup = 1.6, P = 0.22. Group × Time effect: Fgroup×time = 0.3, P = 0.78. Outcome: Worsening depressive symptoms reported after surgery. | NM |
| Park et al 2019 | Global Health Status or QoL Scale, M(SD):  Total:  Baseline: 64.1 (24.7)  12 weeks: 69.3 (21.2)  p=0.06  Stable Disease:  Baseline: 66.3 (22.0)  12 weeks: 70.5 (19.7)  p=0.15  Progressive Disease:  Baseline: 53.6 (35.0)  12 weeks: 67.9 (26.5)  p=0.24. | Physical Functioning M(SD):  Baseline: 78.2 (14.3)  12 weeks: 81.1 (15.7)  p=0.06.  Stable Disease:  Baseline: 77.6 (14.5)  12 weeks: 81.2 (15.5)  p=0.06  Progressive Disease: Baseline: 79.0 (18.2)  12 weeks: 77.1 (20.7)  p=0.63  6MWD (meters, M(SD)):  Stable Disease:  Baseline: 384.2 (74.6)  6 weeks: 426.1 (6.5)  p<.001  12 weeks: 447.4 (50.4)  p<.001  6MWD (Progressive Disease): Unimproved | Fatigue M(SD):  Total:  Baseline: 35.7 (21.2)  12 weeks: 27.1 (22.3)  p<0.001  Stable Disease:  Baseline: 35.0 (22.1)  12 weeks: 26.8 (22.2)  p=0.001  Progressive Disease:  Baseline: 36.5 (24.6)  12 weeks: 34.9 (29.0)  p=0.86  Pain M(SD):  Total:  Baseline: 20.2 (20.9)  12 weeks: 22.9 (23.6)  p=0.33  Stable Disease:  Baseline: 19.0 (21.4)  12 weeks: 25.4 (24.7)  p=0.06  Progressive Disease:  Baseline: 23.8 (16.3)  12 weeks: 14.3 (15.0)  p=0.23 | Anxiety M(SD):  Baseline: 3.9 (4.1)  6 weeks: 3.4 (3.7)  p=0.11  12 weeks: 2.4 (3.8)  p<0.001  Depression M(SD):  Baseline: 4.7 (4.9)  6 weeks: 5.0 (5.2)  p=0.44  12 weeks: 3.5 (4.5)  p=0.02 | Dyspnea M(SD):  Total:  Baseline: 26.7 (23.3)  12 weeks: 25.2 (25.0)  p=0.56  Stable Disease:  Baseline: 27.2 (22.7)  12 weeks: 25.6 (23.4)  p=0.07  Progressive Disease:  Baseline: 19.0 (26.2)  12 weeks: 38.1 (44.8)  p=0.10  Insomnia MSD):  Total:  Baseline: 26.0 (28.2)  12 weeks: 21.3 (28.0)  p=0.12  Stable Disease:  Baseline: 25.1 (29.5)  12 weeks: 22.1 (27.2)  p=0.39  Progressive Disease:  Baseline: 19.0 (32.5)  12 weeks: 28.6 (48.8)  p=0.17  Appetite Loss M(SD):  Total:  Baseline: 21.7 (25.4)  12 weeks: 16.3 (21.5)  p=.047  Stable Disease:  Baseline: 21.5 (24.6)  12 weeks: 14.9 (21.3)  p=.03  Progressive Disease:  Baseline: 33.3 (38.5)  12 weeks: 19.0 (26.2)  p=0.20 |
| Phillips et al 2024 | FACT-G  Fit2ThriveMB: Baseline: 78.9 (SE = 3.3), 12-weeks: 80.8 (SE = 3.3), Change: 2.0 (SE = 1.8); Control: Baseline: 84.0 (SE = 3.4), 12-weeks: 83.1 (SE = 3.4), Change: –0.9 (SE = 1.7), p = 0.25, Cohen’s d = 0.34  Physical Well-being (FACT-B)  Fit2ThriveMB: Baseline: 22.5 (SE = 1.0), 12-weeks: 22.2 (SE = 1.0), Change: –0.3 (SE = 0.6); Control: Baseline: 22.7 (SE = 1.0), 12-weeks: 21.8 (SE = 1.0), Change: –0.8 (SE = 0.6), p = 0.54, Cohen’s d = 0.15 | Physical Function (PROMIS)  Fit2ThriveMB: Baseline: 45.6 (SE = 1.4), 12-weeks: 45.5 (SE = 1.5), Change: –0.1 (SE = 1.3); Control: Baseline: 47.4 (SE = 1.5), 12-weeks: 48.7 (SE = 1.5), Change: +1.4 (SE = 1.3), p = 0.44, Cohen’s d = –0.22  Functional Well-being (FACT-B)  Fit2ThriveMB: Baseline: 19.6 (SE = 1.1), 12-weeks: 20.6 (SE = 1.1), Change: 1.0 (SE = 0.8);  Control: Baseline: 20.4 (SE = 1.1), 12-weeks: 21.2 (SE = 1.1), Change: 0.8 (SE = 0.7), p = 0.85, Cohen’s d = 0.06  SPPB  Fit2ThriveMB: Baseline: 9.4 (SE = 0.4), 12-weeks: 9.8 (SE = 0.4), Change: –0.4 (SE = 0.3);Control: Baseline: 9.7 (SE = 0.4), 12-weeks: 9.2 (SE = 0.4), Change: –0.4 (SE = 0.3), p = 0.06, Cohen’s d = 0.86 | Fatigue( PROMIS)  Fit2ThriveMB: Baseline: 52.7 (SE = 1.8), 12-weeks: 50.0 (SE = 1.8), Change: –2.7 (SE = 1.3); Control: Baseline: 51.3 (SE = 1.8), 12-weeks: 51.9 (SE = 1.8), Change: +0.6 (SE = 1.3). p = 0.08, Cohen’s d = –0.49  Pain Interference (PROMIS)  Fit2ThriveMB: Baseline: 47.9 (SE = 1.8), 12-weeks: 50.8 (SE = 1.9), Change: +2.9 (SE = 1.8); Control:, Baseline: 50.8 (SE = 1.9), 12-weeks: 49.0 (SE = 1.9), Change: –1.8 (SE = 1.8), p = 0.07, Cohen’s d = 0.58 | Anxiety (PROMIS)  Fit2ThriveMB: Baseline: 48.0 (SE = 1.9), 12-weeks: 48.8 (SE = 1.9), Change: +0.8 (SE = 1.2); Control: Baseline: 49.9 (SE = 1.9), 12-weeks: 51.0 (SE = 1.9), Change: +1.1 (SE = 1.2), p = 0.89, Cohen’s d = –0.04  Depression (PROMIS)  Fit2ThriveMB:  Baseline: 46.3 (SE = 1.5), 12-weeks: 46.4 (SE = 1.5), Change: +0.1 (SE = 1.3); Control: Baseline: 46.1 (SE = 1.5), 12-weeks: 46.0 (SE = 1.5), Change: –0.1 (SE = 1.3), p = 0.93, Cohen’s d = 0.01 | Sleep Disturbance (PROMIS)  Fit2ThriveMB: Baseline: 52.1 (SE = 0.8), 12-weeks: 51.8 (SE = 0.9), Change: –0.3 (SE = 0.9); Control: Baseline: 52.0 (SE = 0.8), 12-weeks: 51.4 (SE = 0.8), Change: –0.6 (SE = 0.9), p = 0.80, Cohen’s d = –0.05  Sleep Impairment (PROMIS)  Fit2ThriveMB: Baseline: 49.8 (SE = 1.7), 12-weeks: 47.4 (SE = 1.7), Change: –2.5 (SE = 1.1); Control: Baseline: 49.1 (SE = 1.8), 12-weeks: 47.7 (SE = 1.8), Change: –1.4 (SE = 1.1), p = 0.50, Cohen’s d = –0.19 |
| Purdy et al 2022 | FACT-MM (M(SD)):  Baseline: 111 (23)  12-week: 118 (19)  Change (M(95%CI): 7.3 (0.6, 14.0,  Effect Size: 0.43, MID: NA. | FACT Physical (M(SD)):  Baseline: 20.3 (4.5)  12-week: 20.9 (4.0)  Change (M(95%CI)): 0.6 (−1.0, 2.2),  Effect Size: 0.16, MID: 2–3  FACT Functional (M(SD)):  Baseline: 17.2 (4.3)  12-week: 17.9 (4.2)  Change (M(9%CI)): 0.7 (−0.6, 2.0)  Effect Size: 0.21, MID: 2–3  Physical Assessments:  2-min Step Test (steps; M(SD)):  Baseline: 68.6 (17.7)  12-week: 81.3 (16.0)  Change (M(95%CI)): 12.7 (8.7, 16.8)  Effect Size: 1.28, MID: NA  30-s Sit-to-Stand (reps; M(SD)):  Baseline: 13.1 (4.5)  12-week: 15.8 (4.3)  Change (M(95%CI)): 2.7 (1.6 to 3.8)  Effect Size: 1.00, MID: >2  Plank Duration (sec; M(SD)):  Baseline: 78.3 (46.0)  12-week: 119.9 (± 73.4)  Change (M(95%CI)): 41.6 [CI: 22.3 to 60.8], Effect Size: 0.98, MID: NA.  Timed Single-Leg Stance Test (sec; M(SD)):  Baseline: 23.1 (13.3)  12-week: 31.8 (12.6)  Change (M(95%CI)): 8.7 (4.6, 12.8)  Effect Size: 0.86, MID: 24  Active Shoulder Flexion (degrees; M(SD)):  Baseline: 146.8 (11.7)  12-week: 149.4 (11.5)  Change (M(95%CI)): 2.6 (0.7 to 4.5)  Effect Size: 0.56, MID: > 10  Modified Sit-and-Reach (cm; M(SD)):  Baseline: -5.8 (± 14.0)  12-week: -2.2 (± 13.6)  Change (M(95%CI): 3.6 (1.6, 5.7)  Effect Size: 0.81, MID: NA. | FACT-Fatigue (M(SD)): Baseline: 35 (10)  12-week: 37 (8)  Change (M(95%CI)): 1.7 (−1.4 to 4.8)  Effect Size: 0.21, MID: 3 | FACT emotional (score). Baseline: 16.4 (4.9)  12-week: 18.5 (3.1)  Change (M(95%CI)): 2.1 (0.6 to 3.6). Effect size: 0.57. MID: 2-3. | NM |
| Schmitz et al 2021 | NM | NM | Global Fatigue (M(SD)):  Immediate Start:  Month 0: 2.7 (1.9)  Month 3: 2.5 (0.9)  SMD: -0.10  Delayed Start:  Month 0: 3.0 (2.2)  Month 3: 3.3 (2.0)  SMD: 0.14  Hedges’ g (95% CI): 0.24 (−0.70, 1.17)  Pain Severity (M(SD):  Immediate Start:  Month 0: 7.9 (5.3)  Month 3: 7.6 (5.6)  SMD: -0.05  Delayed Start:  Month 0: 5.4 (7.0)  Month 3: 5.0 (4.4)  SMD: -0.06  Hedges’ g (95% CI): −0.03 (−0.96 to 0.90)  Pain Interference (M(SD)):  Immediate Start:  Month 0: 10.9 (11.7)  Month 3: 9.1 (10.0)  SMD: -0.15  Delayed Start:  Month 0: 10.0 (9.5)  Month 3: 9.14 (10.32)  SMD: -0.09  Hedges’ g (95% CI): 0.08 (−0.85 to 1.01) | Distress (M(SD)):  Immediate Start:  Month 0: 10.0 (10.3)  Month 3: 6.1 (5.6)  SMD: 0.38  Delayed Start:  Month 0: 9.6 (7.5)  Month 3: 12.86 (14.5)  SMD: -0.44  Hedges’ g (95% CI): 0.74 (−0.31 to 1.79) | Pittsburgh Sleep Quality Index (M(SD)):  Immediate Start:  Month 0: 8.8 (3.5)  Month 3: 12.6 (3.3)  SMD: 1.10  Delayed Start:  Month 0: 7.9 (3.5)  Month 3: 14.1 (3.5)  SMD: 1.78  Hedges’ g (95% CI): 0.65 (−0.30 to 1.61) |
| Schmitz et al 2023 | SF-36 General Health (M(SD)):  Immediate Tx:  Baseline: 54.52 (19.03)  3-month change: 0.76 (11.98)  Delayed Tx:  3-month: 55.50 (17.98)  6-month change: −1.67 (9.39)  Control:  Baseline:54.76 (18.81)  3-month change: −0.50 (12.97)  Immediate vs. Control p=0.7567  Delayed vs. Control p=0.5029 | Chair Stands (sec, M(SD)):  Immediate Tx:  Baseline: 11.29 (3.85)  3-month change: −1.16 (2.91)  Delayed Tx:  3-month: 11.06 (3.52)  6-month change −0.97 (3.50)  Control:  Baseline: 11.62 (3.82)  3-month change: −0.58 (3.29)  Immediate vs. Control p=.5718  Delayed vs. Control p=.2998  SF-36 Physical Functioning M(SD):  Immediate Tx:  Baseline: 71.64 (24.77)  3-month change: 8.06 (19.03)  Delayed Tx:  3-month value: 70.75 (22.14)  6-month change −1.00 (14.29)  Control:  Baseline: 70.00 (22.14)  3-month change 0.00 (10.39)  Immediate vs. Control p=.1230  Delayed vs. Control p=.7904 | SF-36 Pain (M(SD)):  Immediate Tx:  Baseline: 62.50 (22.30)  3-month change: 3.61 (19.18)  Delayed Tx:  3-month value: 64.00 (22.69)  6-month change 2.17 (14.20)  Control:  Baseline: 65.00 (22.05)  3-month change: −2.00 (18.65)  Immediate vs. Control p=.3677, Delayed vs. Control p=.5640  Pain Severity (0–10; M(SD)):  Immediate Tx:  Baseline: 3.57 (2.73)  3-month change: 0.33 (2.70)  Delayed Tx:  3-month value: 3.35 (2.89)  6-month change −1.81 (2.68)  Control:  Baseline: 3.57 (2.93)  3-month change: −0.10 (2.02)  Immediate vs. Control p=0.5829, Delayed vs. Control p=.0204  Pain Interference (0–10; M(SD)):  Immediate Tx:  Baseline: 2.06 (2.17)  3-month change: −0.60 (0.91)  Delayed Tx:  3-month value: 1.44 (1.48)  6-month change 1.40 (1.53)  Control:  Baseline: 2.49 (2.67)  3-month change −0.86 (2.46)  Immediate vs. Control p=.6713, Delayed vs. Control p=.0333  SF-36 Vitality/Fatigue M(SD):  Immediate Tx:  Baseline: 49.76 (21.12)  3-month change: 6.67 (12.83)  Delayed Tx:  3-month value: 51.25 (17.61)  6-month change: 0.00 (16.80)  Control:  Baseline: 49.05 (18.41)  3-month change: 2.00 (17.35)  Immediate vs. Control p=.3494, Delayed vs. Control p=1.0000 | NM | Sleep Disturbance Score (0–100, M(SD))  Immediate Tx:  Baseline: 53.12 (2.92)  3-month change: 0.17 (3.88)  Delayed Tx:  3-month value: 52.89 (3.68)  6-month change −0.78 (3.54)  Control:  Baseline: 54.04 (3.53)  3-month change −0.96 (3.16)  Immediate vs. Control p=.3325, Delayed vs. Control p=.4077 |
| Shachar et al 2023 | PROMIS Global Health:  Baseline: 43.9 (10.9)  Three months: 45.6 (11.2)  p=0.49  FACT-General M(SD):  Baseline: 82.2 (16.3)  Three months: 83.9 (17.7)  p=0.25 | PROMIS Physical Function (M(SD)):  Baseline: 46.3 (8.6)  Three months: 46.0 (8.3)  p=0.97  FACT_G Physical Well-Being M(SD):  Baseline: 21.1 (5.5)  Three months: 21.5 (6.0)  p=0.36  Functional Well-Being M(SD):  Baseline: 19.2 (5.6)  Three months: 20.1 (5.6)  p=0.11  PSEFSM M(SD):  Baseline: 7.3 (2.3)  Three months: 7.2 (2.3)  p=0.97  Physical Activity (minutes/week, M(SD)):  Baseline: 124.2 (134.7)  Three months: 170.9 (140.7)  p=0.04 | PROMIS Pain Interference M(SD):  Baseline: 50.8 (9.4)  Three months: 50.2 (8.0)  p=0.60  PROMIS Fatigue M(SD):  Baseline: 53.5 (9.2)  Three months: 53.7 (10.0)  p=0.63  FACIT-Fatigue M(SD):  Baseline: 35.7 (12.1)  Three months: 37.0 (10.8)  p=0.31 | PROMIS Depression M(SD):  Baseline: 48.3 (8.6)  Three months: 46.4 (8.6)  p=0.09  PROMIS Anxiety M(SD):  Baseline: 52.2 (8.8)  Three months: 48.5 (8.6)  p=0.02  PROMIS Mental Health M(SD):  Baseline: 50.2 (6.9)  Three months: 50.6 (6.7)  p=0.78 | PROMIS Sleep Quality M(SD):  Baseline: 52.5 (8.2)  Three months: 51.4 (7.8)  p=0.18 |
| Soh et al 2018 | NM | NM | NM | NM | NM |
| Wallace et al 2025 | T0 – T1 Group Effect (CM-CL): 0.03 [-0.08, 0.13], p=0.062 | NM | NM | NM | NM |
| Wang et al 2021 | FACT M(SD):  Baseline: 3.1 (0.1)  Week 6: 3.1 (0.1)  Mean Difference: 0, Cohen's d: 0  MD Anderson Symptom Inventory M(SD):  Baseline: 3.4 (1.1)  Week 6: 3.7 (2.6)  Mean Difference: -0.3, Cohen's d: 0.2  Lawton Instrumental Activity of Daily Living Scale M(SD):  Baseline: 29.8 (1.9)  Week 6: 30.0 (2.0)  Mean Difference: -0.2, Cohen's d: 0.1 | Preferred Gait Speed (m/s, M(SD)):  Baseline: 0.9 (0.2)  Week 3: 1.0 (0.3)  Week 6: 1.1 (0.3)  Mean Difference: -0.2, Cohen's d: 0.8  6-Min Walk Test (meters, M(SD)):  Baseline: 344.1 (78.5)  Week 3: 381.4 (88.1)  Week 6: 394.2 (99.7)  Mean Difference: -50.1, Cohen's d: 0.6  Combined Grip Sum (kg, M(SD)):  Baseline: 0.6 (0.2)  Week 3: 0.6 (0.2)  Week 6: 0.6 (0.2)  Mean Difference: 0, Cohen's d: 0 | Brief Fatigue Inventory (M(SD)):  Baseline: 5.4 (2.0)  Week 6: 5.7 (3.0)  Mean Difference: -0.3, Cohen's d: 0.1  Worst Fatigue (M(SD)):  Baseline: 8.0 (0.8)  Week 6: 6.3 (3.5)  Mean Difference: 1.8. Cohen's d: 0.7  Brief Pain Inventory (M(SD)):  Baseline: 3.7 (2.5)  Week 6: 3.3 (3.7)  Mean Difference: 0.4, Cohen's d: 0.1  Worst Pain (M(SD)):  Baseline: 7.3 (1.0)  Week 6: 5.5 (4.2)  Mean Difference: 1.8, Cohen's d: 0.6 | NM | NM |
| Wolff et al 2024 | PHQ-9 Baseline Score: IG MBC: 9.7, Overall Cohort: 7.9, Change from Baseline to T3 (Δ): –1.3  95% Confidence Interval (CI): [–0.56, 4.82], P-value: 0.12, Relative Reduction: 13.4% decrease in PHQ-9 total score from baseline | NM | NM | NM | NM |
| Note: MMRC: Medical Research Council; NS: Non-Significant; MID: minimally important difference; NA: not applicable; PSEFSMP: Perceived Self-Efficacy for Fatigue Self-Management PROMIS: The Patient-Reported Outcomes Measurement Information System; QALYs: Quality Adjusted Life Years; IQR: Inter-quartile range; VAS: Visual Analouge Scale; ABC Scale: Activities-Specific Balance Confidence Scale; AM-PAC-CAT: Activity Measure for Postacute Care Computer-Adaptive Test; EQ-5D-3L: EuroQol 5-Dimension 3-Level; FACT-G: Functional Assessment of Cancer Therapy – General; NM: Not measured; IG: Intervention Group; UC: Usual Care; EORTC QLQ-C30: European Organisation for Research and Treatment of Cancer Quality of Life Questionnaire; PHQ: Patient Health Questionnaire; ECOG: Eastern Cooperative Oncology Group Performance Status; METs: Metabolic Equivalent of Tasks; ES: Effect size. | | | | | |
